# SARS-CoV-2 early infection signature identified potential key infection mechanisms and drug targets

**DOI:** 10.1186/s12864-021-07433-4

**Published:** 2021-02-18

**Authors:** Yue Li, Ashley Duche, Michael R. Sayer, Don Roosan, Farid G. Khalafalla, Rennolds S. Ostrom, Jennifer Totonchy, Moom R. Roosan

**Affiliations:** 1 School of Pharmacy, Chapman University, Irvine, CA 92618 USA; 2 College of Pharmacy, Western University of Health Sciences, Pomona, CA 91766 USA; 3 College of Pharmacy, California Health Sciences University, Clovis, CA 93612 USA

**Keywords:** COVID-19, SARS-CoV-2 gene expression signature, Bronchoalveolar lavage fluid cells, Peripheral blood mononuclear cells, COVID-19 treatments

## Abstract

**Background:**

The ongoing COVID-19 outbreak has caused devastating mortality and posed a significant threat to public health worldwide. Despite the severity of this illness and 2.3 million worldwide deaths, the disease mechanism is mostly unknown. Previous studies that characterized differential gene expression due to SARS-CoV-2 infection lacked robust validation. Although vaccines are  now available, effective treatment options are still out of reach.

**Results:**

To characterize the transcriptional activity of SARS-CoV-2 infection, a gene signature consisting of 25 genes was generated using a publicly available RNA-Sequencing (RNA-Seq) dataset of cultured cells infected with SARS-CoV-2. The signature estimated infection level accurately in bronchoalveolar lavage fluid (BALF) cells and peripheral blood mononuclear cells (PBMCs) from healthy and infected patients (mean 0.001 vs. 0.958; *P* < 0.0001). These signature genes were investigated in their ability to distinguish the severity of SARS-CoV-2 infection in a single-cell RNA-Sequencing dataset. *TNFAIP3*, *PPP1R15A*, *NFKBIA*, and *IFIT2* had shown bimodal gene expression in various immune cells from severely infected patients compared to healthy or moderate infection cases. Finally, this signature was assessed using the publicly available ConnectivityMap database to identify potential disease mechanisms and drug repurposing candidates. Pharmacological classes of tricyclic antidepressants, SRC-inhibitors, HDAC inhibitors, MEK inhibitors, and drugs such as atorvastatin, ibuprofen, and ketoconazole showed strong negative associations (connectivity score < − 90), highlighting the need for further evaluation of these candidates for their efficacy in treating SARS-CoV-2 infection.

**Conclusions:**

Thus, using the 25-gene SARS-CoV-2 infection signature, the SARS-CoV-2 infection status was captured in BALF cells, PBMCs and postmortem lung biopsies. In addition, candidate SARS-CoV-2 therapies with known safety profiles were identified. The signature genes could potentially also be used to characterize the COVID-19 disease severity in patients’ expression profiles of BALF cells.

**Supplementary Information:**

The online version contains supplementary material available at 10.1186/s12864-021-07433-4.

## Background

The 2019 coronavirus pandemic (COVID-19), caused by the novel Severe Acute Respiratory Syndrome Coronavirus 2 (SARS-CoV-2), has already contributed to over 107 million confirmed cases and 2.3 million deaths worldwide [1]. The Centers for Disease Control and Prevention (CDC) has developed test kits to diagnose the SARS-CoV-2 virus RNA from nasopharyngeal (NP) or oropharyngeal (OP) swabs using real-time reverse transcription polymerase chain reacton  (RT-PCR) [2, 3]. However, the detection of SARS-CoV-2 RNA was shown to be much higher with NP swabs than OP swabs, 63% compared to 32%, respectively [4]. Therefore, sputum or BALF may be better suited for the detection of the SARS-CoV-2 virus due to the high viral load observed in BALF [5]. Despite previous advancements in our knowledge of SARS-CoV-2, significant gaps still exist within our understanding of COVID-19 and clinical care, such as the uncertainty of mortality risk in critically ill patients. However, publicly available studies and datasets can be further leveraged to learn more about COVID-19 pathophysiology and treatment [6].

Beyond diagnostic procedures, understanding the mechanisms of action to begin the formulation of potential drug therapies is crucial. Previous studies have shown that SARS-CoV-2 infection begins with SARS-CoV-2 viral entry through a host receptor, angiotensin-converting enzyme 2 (*ACE2*) [7]. The cellular serine protease *TMPRSS2* is also a susceptibility factor since it primes the spike protein of SARS-CoV-2 [8, 9]. *ACE2* and *TMPRSS2* are primarily expressed in bronchial transient secretory cells, nasal and mouth tissues [9, 10]. Therefore, drug therapies inhibiting SARS-CoV-2 interaction with *ACE2 or TMPRSS2* may be promising for COVID-19 treatments. On the other hand, up-regulation of *ADAM17* has been shown to leads to the *ACE2* ectodomain proteolytic cleavage in which regulation of the *ADAM17/ACE2* axis may be a potential target by treatments such as paricalcitol, a synthetic vitamin D analog [11, 12]. Additional drug target therapies have also been proposed, such as recombinant soluble *ACE2*, indirect *ACE2* modulators (angiotensin receptor blockers, calmodulin antagonists, selective estrogen receptor modifiers), *TMPRSS2* inhibitors (camostat, nafamostat, antiandrogens, inhaled corticosteroids), and *ADAM-17* enhancers (5-fluorouracil) [12]. Since drug development and approval of a new treatment is a critically lengthy process to ensure safety and effectiveness, repurposing currently available drugs with known safety profiles is a lucrative strategy. Initially, a few repurposed drugs, including chloroquine, hydroxychloroquine, lopinavir/ritonavir, ribavirin, oseltamivir, were thought to be promising. However, there is a lack of strong evidence supporting the effectiveness of these therapies against COVID-19 [13–15]. Although intravenous remdesivir has now been approved by the US Food and Drug Administration (FDA) due to its proven efficacy in multiple clinical trials in reducing critically ill COVID-19 patients recovery time by 5 days, effective treatment options are limited [15, 16]. As adjunctive therapy, supporting evidence of the role of corticosteroid in COVID-19 treatment has also been inconsistent. The Randomized Evaluation of COVid-19 thERapY (RECOVERY) trial has shown a significant reduction of death by 35% in ventilated patients and 20% in patients on supplemental oxygen therapy with dexamethasone in severe cases [17]. Further advancements are currently under investigation in clinical trials underway with many antivirals, anti-cytokines, immunomodulatory, and immunoglobulin agents as COVID-19 treatment to improve current therapies [13].

Gene expression signatures, representing transcriptional activities of a disease or biological phenomenon, can be utilized to potentially identify novel drug targets for COVID-19. This method has been applied to characterize many conditions, including cancer effectively, and used to identify potential treatments for many years [18, 19]. Essentially, gene expression signatures consist of the most discriminatory differentially expressed genes for a disease or biological phenomenon. Application of gene expression signatures has been used for viral infection or severity of infection assessment. Researchers have developed virus infection signature of dengue and other viruses to assess severity of infection, secondary infection, reservoirs in hosts, or origin of “orphan viruses” [20, 21]. Differentially expressed genes (DEGs) studies have also been conducted for SARS-CoV-2 infection [22]. However, these DEGs studies were not robustly validated in independent datasets or different cell types with SARS-CoV-2 infections.

In this study, we sought to characterize the transcriptional response to SARS-CoV-2 infection by generating a gene expression signature, a set of genes representing infection in the host that can be used as a surrogate measure of the infection-related transcriptional activity, using a publicly available dataset derived from infecting cultured cells with SARS-CoV-2 [19, 23–26]. The gene signature was then validated in independent datasets (CRA002390, SRR10571724, SRR10571730, and SRR10571732) from COVID-19 patients, specifically in BALF cell and peripheral blood mononuclear cell (PBMC) samples [22]. The signature genes were also investigated in a single-cell RNA-Sequencing (scRNA-Seq) dataset (GSE145926) to evaluate the role of genes’ expression in COVID-19 disease severity [27]. Finally, the signature genes were assessed for similar perturbations and potential drug targets by using ConnectivityMap (CMAP) database.

## Results

### Signature generation and validation

**Fig. 1:**
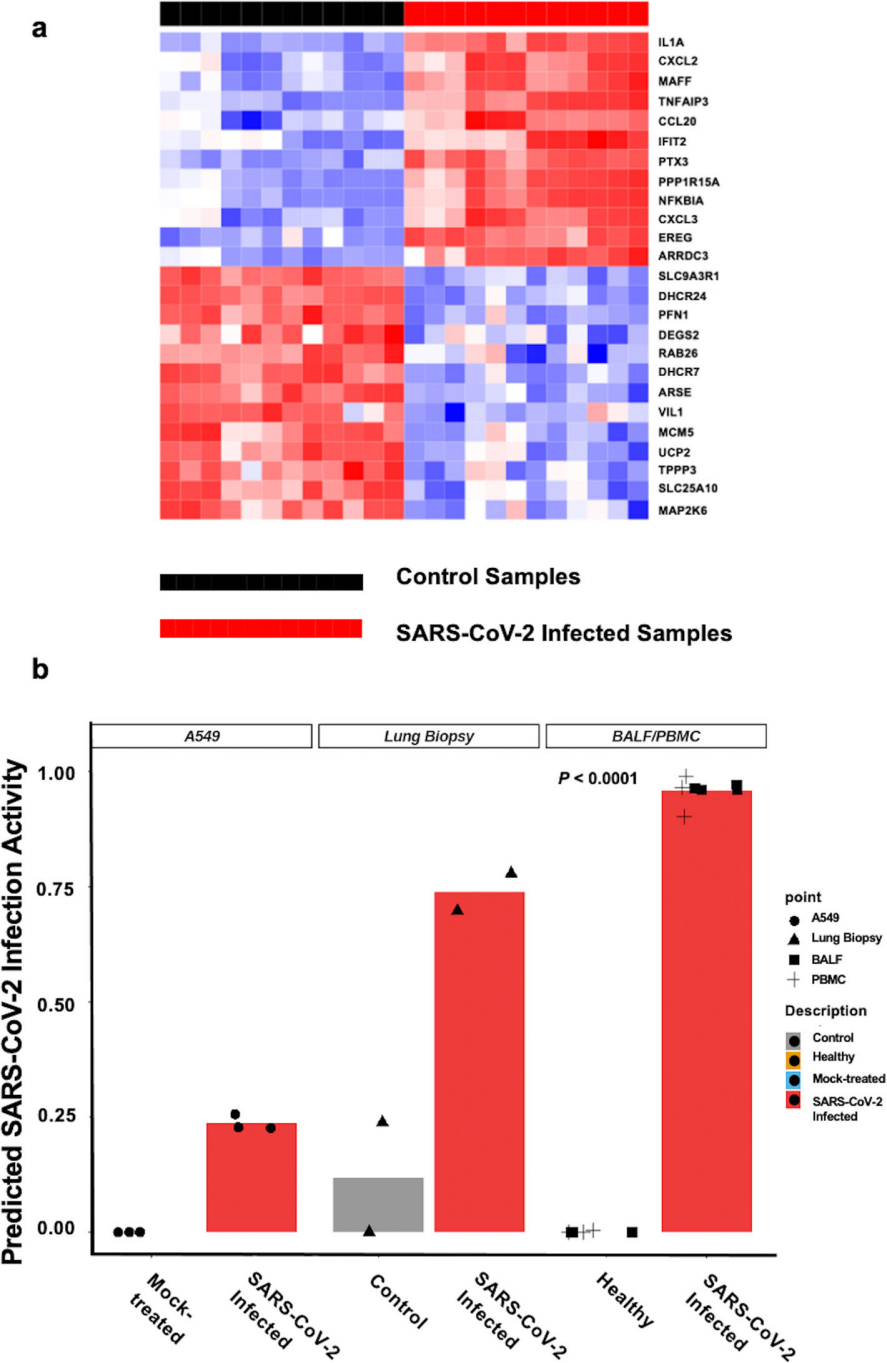
Development and validation of the 25-gene SARS-CoV-2 signature. **a** 25 gene SARS-CoV-2 signature using cell lines A549 overexpressed with *ACE2* and Calu-3 infected with SARS-CoV-2 (Multiplicity of infection [MOI] 2) compared to mock-treated. **b** Internal and external validation of SARS-CoV-2 infection activity by using the signature in series 2, 15, bronchoalveolar lavage fluid (BALF) cells (*n* = 7) and peripheral blood mononuclear cells (PBMC) (*n* = 6) samples. Series 2 consisted of A549 cells infected with mock or SARS-CoV-2 (MOI 0.2), whereas series 15 consisted of postmortem COVID-19 patients and healthy lung biopsy samples. BALF cells and PBMC were collected from healthy and SARS-CoV-2 infected patients

To develop a gene expression signature representative of COVID-19, a computational analysis tool known as *Adaptive Signature Selection and InteGratioN* (ASSIGN) was used on cell lines infected with SARS-CoV-2 (GSE147507). An optimal SARS-CoV-2 infection signature of 25 genes was generated consisting of 12 upregulated and 13 downregulated genes (Table 1, Fig. 1a). Genes that showed the highest discrimination between the control and SARS-CoV-2 infected training samples were selected. Leave-one-out-cross-validation (LOOCV) plot demonstrated an internal validity of the signature displaying infection activity of the samples. The 12 samples infected with SARS-CoV-2 showed high infection activity, while the control samples showed no infection activity (Fig. S1). Ingenuity Pathway Analysis (IPA) revealed that ‘Interferon Signaling’ and ‘Role of Pattern Recognition Receptors in Recognition of Bacteria and Viruses’ pathways were significantly enriched for genes differentially expressed in SARS-CoV-2 infected cell lines compared to mock-treated cells (*P-value* = 2.37 × 10^− 13^ and 7.37 × 10^− 11^, respectively; Fig. S2).

**Table 1:** 25-gene SARS-CoV-2 infection signature. The 25-gene SARS-CoV-2 infection signature listing the genes with positive and negative weights indicating upregulated and downregulated expression, respectively. Twelve genes are upregulated and 13 genes are downregulated in the SARS-CoV-2 infection compared to control samples in the signature

Gene Symbol	Weight
*IL1A*	5.094452868
*CXCL2*	4.167754294
*TNFAIP3*	3.979661316
*MAFF*	4.005425324
*PPP1R15A*	3.656610829
*NFKBIA*	3.65420358
*PTX3*	3.830000483
*CXCL3*	3.592261061
*CCL20*	3.84337845
*IFIT2*	3.837096394
*ARRDC3*	3.454679035
*EREG*	3.483351558
*ARSE*	−1.826349018
*MAP2K6*	−2.116193528
*DHCR7*	−1.780337716
*UCP2*	−1.924820651
*SLC25A10*	−1.98531707
*VIL1*	−1.840480348
*MCM5*	−1.918251715
*DHCR24*	−1.637659814
*SLC9A3R1*	−1.532035311
*PFN1*	−1.63976461
*TPPP3*	−1.952896573
*DEGS2*	−1.652290762
*RAB26*	−1.7067497

Next, the gene signature was further tested in two series from the same GSE147507 dataset for additional internal validation (Fig. 1b). Series 2 contained A549 cells with mock or SARS-CoV-2 (Multiplicity of Infection MOI, 0.2) infection, while series 15 contained lung samples from postmortem otherwise healthy or COVID-19 patients Series 2 contained A549 cells that were not well-infected with SARS-CoV-2 virus [26]. The signature detected higher infection activity in the infected cell lines than in control samples as well as predicted high infection activity in lung biopsy samples from patients with COVID-19 and no infection in healthy samples. In low-level SARS-CoV-2 infected A549 cells, the signature detected higher infection in the infected samples but at a lower level than the lung biopsy with COVID-19 patient samples. Thus, the 24-hour post-infection SARS-CoV-2 signature accurately predicted infection status during internal validation in the postmortem lung samples from COVID-19 patients and cell lines with very low SARS-CoV-2 infection.

Finally, the 25-gene signature was validated in an independent external validation dataset with seven BALF cells and six PBMC samples from COVID-19 patients (CRA002390, SRR10571724, SRR10571730, and SRR10571732; Fig. 1b). All infected patients’ samples were predicted to have higher infection activity compared to healthy control samples (mean predicted activity: 0.958 vs. 0.001; *P* < 0.0001). Thus, the signature was internally tested and then further validated in an external independent dataset from multiple COVID-19 patient samples.

### Expression patterns of a gene signature in scRNA-Seq

**Fig. 2:**
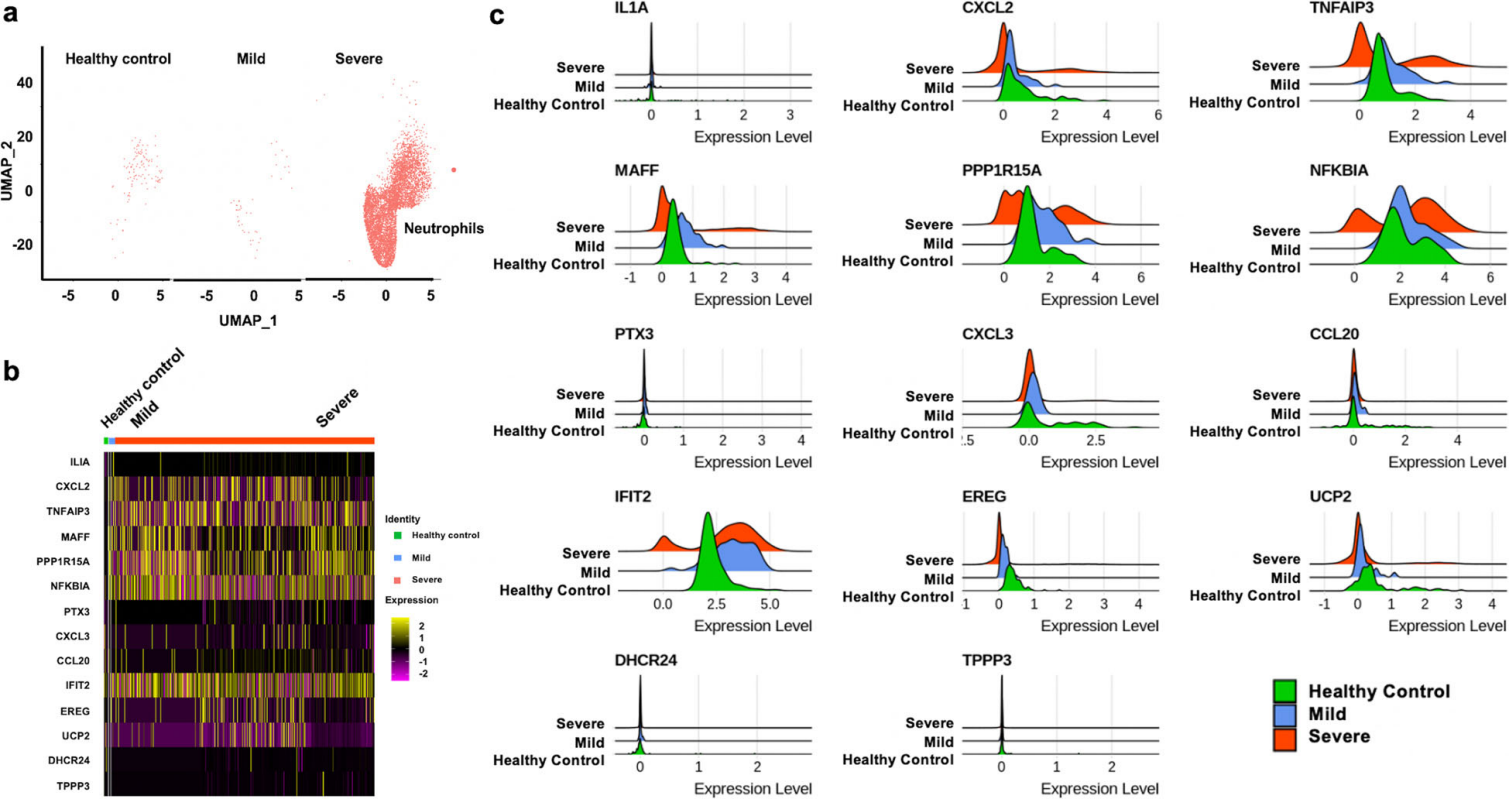
Expression of signature genes in neutrophils from respective patient groups. **a** Uniform Manifold Approximation and Projection (UMAP) plots of the neutrophils. Each dot corresponds to one single cell. **b** Heatmap of fourteen signature genes in three groups. Each vertical bar represents a single cell. Column (cell identity) width is proportional to the number of cells present in that cluster. **c** Distributions of signature gene expression shown in ridge plots. Red, blue and green colors represent gene expression values in severe, mild patients and healthy controls, respectively

Following signature validation, the signature genes were evaluated in scRNA-seq data (GSE145926) to assess their roles in SARS-CoV-2 infection severity. The signature genes were investigated in BALF cells from six patients with severe COVID-19 disease, three patients with moderate COVID-19 disease, and three healthy controls. Using cell markers, Uniform Manifold Approximation and Projection (UMAP) clustering analysis, eight types of cells were identified, including macrophages, basal cells, dendritic cells, naïve CD4+ T cells, neutrophils, natural killer (NK) cells, plasma cells, and T cells (Table S1, Fig. S3). Higher counts of neutrophils, basal and dendritic cells were found in BALF cells from severe COVID-19 patients compared to healthy controls (Fig. 2a, Fig. S4-S10a). In basal, dendritic, and T cells, *CXCL2, TNFAIP3, MAFF, PPP1R15A, NFKBIA* showed higher expression levels in severely infected patients than mildly infected patients and healthy controls (Fig. 2b, and Fig. S4-S10b).

To explore the feature distributions of the signature genes in infected patients and healthy controls, ridge plots were studied for the 14 genes shared between the signature and the sc-RNA dataset. (Fig. 2c and Fig. S4-S10c). In general, the gene expression distributions were similar in mildly infected patients and healthy controls compared to severely infected patients. Some signature genes showed differential expression in various immune cells and may indicate the severity of the infection. Specifically, among the upregulated signature genes, *TNFAIP3, PPP1R15A, NFKBIA, IFIT2* had bimodal gene expression distributions in the immune cells from severely infected patients compared to healthy or mildly infected patients, while the chemokine genes or chemokine inducible genes, *IL1A, CXCL2, CXCL3, CCL20,* and *PTX3* showed minimal variance. Compared to other cells, *IFIT2* had lower expression levels in a majority of the plasma cells from patients with severe disease compared to patients with moderate disease and healthy controls. Among the downregulated signature genes, *UPC2* showed slightly decreased expression in dendritic cells, macrophages, and NK cells from patients with severe disease compared to healthy or infected individuals with moderate COVID-19 disease. *DHCR24* and *TPPP3* genes showed limited to no variance in the infection severity.

### Analysis of signature genes for perturbagen evaluation

**Fig. 3:**
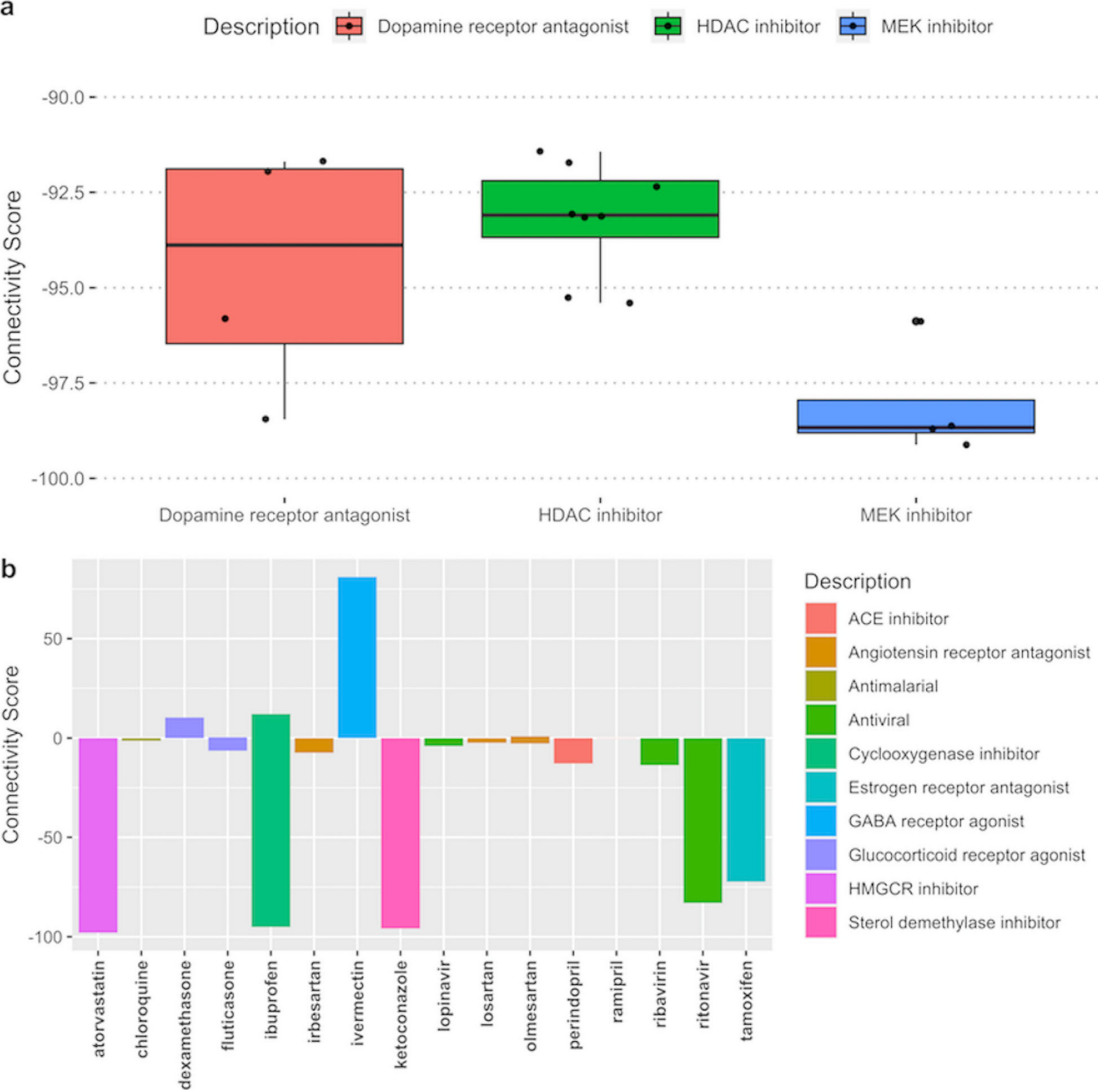
Pharmacologic signature connections identified in the ConnectivityMap (CMAP) database. **a** Distribution of strong connectivity scores (CS) for the top three pharmacologic classes with 4 or more compounds. **b** Bar plot of individual pharmacologic compounds. Positive CSs indicate the degree of similarity and negative CSs indicate the degree of dissimilarity. -90 > = CS or CS > = 90 was considered strong dissimilar and similar connections, respectively

To characterize the patterns of SARS-CoV-2 transcriptional activity in existing datasets, a gene expression query was performed using these 25 genes in the CMAP database. There were 493 strong connections with the 25-gene signature in the CMAP database characterized by connectivity scores (CS), of which 45 were treatments with various pharmacologic compounds. Genetically, the SARS-CoV-2 infection signature was most alike in conditions where *NFkB* was activated via overexpression of various tumor necrosis factor receptor family genes (CS 99.9), knockdowns of heat shock proteins, and vesicular transport (CSs 97.7 and 96, respectively). Knockdowns of *SYPL1, NDUFB6, RYBP*, multiple G-protein coupled receptors (GPCRs), including purinergic receptor *P2RY2*, multiple CD molecules, *PRPF4, IL8, RPIA, TAF15, PCGF3, LSS, CXCL2,* and *CCDN2,* were strongly negatively (CS < − 95) connected with the signature. Pharmacologically, MEK inhibitors, SRC inhibitors, and tricyclic antidepressants (TCAs) were found to have the most opposing signature to the SARS-CoV-2 infection signature (CSs − 98.7, − 95.1 and − 92, respectively). These drugs may oppose the effects of SARS-CoV-2 viral infection. Many HDAC inhibitors, growth-factor targeting drugs, dopamine receptor inhibitors, ibuprofen, ketoconazole, chromamycin-a3, and atorvastatin showed strong negative connections (Fig. S11, Fig. 3), suggesting these drugs may have a modulating effect in SARS-CoV-2 infection. Additionally, CSs were also composed of other potential drugs available in the CMAP database that are currently or were previously considered for COVID-19 treatments, including chloroquine, ribavirin, angiotensin-converting enzyme (ACE) inhibitors / angiotensin receptor blockers (ARB), lopinavir, dexamethasone, and other glucocorticoids. None of these had a strong connection with our query signature (Table S2). The antiviral with the strongest negative connection was ritonavir (CS − 82.9).

## Discussion

**Fig. 4:**
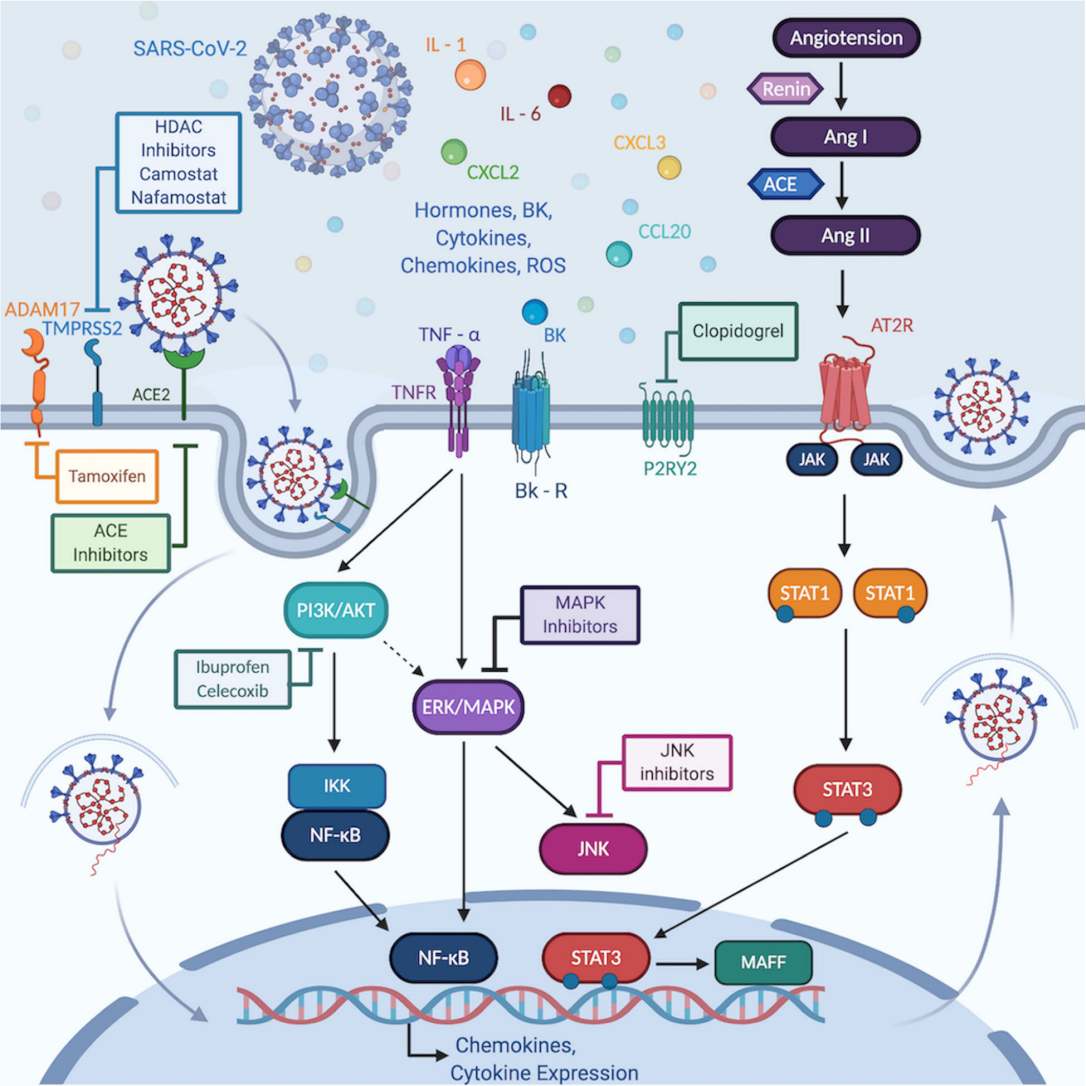
Potential SARS-CoV-2 infection mechanism influencing potential drug choices for repurposing. The SARS-CoV-2 virus enters the cells through *ACE2* receptors facilitated by *TMPRSS2 and ADAM17.* Drug molecules inhibiting the *ACE2/TMPRSS2* axis dampen viral entry into the cell. Angiotensin II also activates *JAK/STAT* pathways upregulating proinflammatory cytokines. *IL-1*, *TNF-α* cytokines are mediators of innate immunity to stimulate an early innate response. These cytokines activate growth factor receptor pathways, such as *PI3K/AKT* and *MAPK* pathways leading to increased proinflammatory cytokines production via the *NF-kB* transcription factor. Therefore, JAK/STAT, PI3K/AKT and MAPK inhibitors may be beneficial in preventing inappropriate immune response. Inflammatory chemokines such as *CXCL2*, *CXCL3*, *CCL20* attract other immune cell types to fight the infection and repair tissue damage leading to local tissue inflammation and cytokine storm. Glucocorticoids may help immune response associated with cytokine storm. G-protein coupled receptors, including bradykinin receptors and purinergic receptors, are also associated with SARS-CoV-2 infection

Infection of the SARS-CoV-2 virus can wreak havoc on the body and cause severe pulmonary disease. Currently, we lack an adept understanding of disease mechanisms and effective drug therapy for this fatal disease [22]. As new variants continue to emerge, the scientific community and healthcare officials are racing to find effective COVID-19 treatments and vaccines. A gene expression signature capable of effectively characterizing the host transcriptional activity resulting from the infection can be translated to a biomarker for treatment selection. Multiple publicly available datasets were leveraged and a flexible Bayesian factor analysis approach was used to develop and validate a SARS-CoV-2 infection signature consisting of 12 upregulated and 13 downregulated genes. These genes were profiled in single cells obtained from BALF cells of healthy and infected patients to assess transcriptional variance in disease severity. Furthermore, the signature was applied to CMAP, a publicly available gene expression signature database, to identify drugs that oppose this signature and could serve as potential drug candidates for treating SARS-CoV-2 infection. Finally, the SARS-CoV-2 infection mechanism influencing potential drug choices for repurposing was proposed (Fig. 4).

From our current understanding, the mechanism of action for SARS-CoV-2 viral entry is that the virus enters the cells through *ACE2* receptors facilitated by *TMPRSS2* spike proteins and activates the renin-angiotensin (RAS) system. The RAS system controls many critical aspects of the circulatory system, including bradykinin (BK) regulation of blood pressure. Current evidence suggests that a subgroup of patients with severe COVID-19 may experience “cytokine storm” syndrome indicating an extreme host immune response [15, 28]. Targeting *IL-6, IL-1,* and *JAK/STAT* protein can be used as approaches to suppress the cytokine storm [29]. Other studies propose an alternate theory in COVID-19, a “bradykinin storm.” The bradykinin storm theory can explain many of the symptoms of COVID-19. Angiotensin-converting enzyme (*ACE*) typically degrades BK, but the SARS-CoV-2 virus downregulates *ACE*. Thus, more BK remains active. As BK builds up, so does the vascular permeability. As a result, the lungs fill with fluid, and immune cells leak into the lungs, causing severe inflammation.

Bradykinin receptors are GPCRs and are known for their role as proinflammatory mediators [30]. Proinflammatory mediators such as chemokines (*CXCL2*, *CXCL3*, *CCL20*), BK, tumor necrosis factors, and interleukins stimulate GPCRs and activate intracellular *MAPK, NF-kB*, and *MAFF* dependent inflammatory pathways [31]. *IFIT2* expression has also been shown to induce proinflammatory cytokine response both in vitro and in vivo. Activation of the *MAPK/NF-kB* signaling pathway, in turn, upregulate airway kinin receptors leading to airway hyperreactivity [32]. Knockdowns of other GPCRs*,* including *GPR137, GPR65*, purinergic receptor *P2RY2,* were strongly negatively connected with the signature, indicating potential interaction with inflammatory pathways and platelet adhesion [33]. Therefore, the roles of these *GPCRs* need to be further investigated in COVID-19.

Among the other signature genes, upregulation of *NFKBIA* has been associated with the survival, activation, and differentiation regulation of immune cells [34]. ACE2 mediated activation of *ACE/AngII/AT1R* axis leading to hyperactivation of *NFKBIA*, ultimately precipitating cytokine storm in COVID-19 patients [35]. Under the normal physiologic condition, *ACE/Ang II/AT1R* axis activation is compensated by *Ang-(1–7)* and downregulation of the *NFKBIA* expression [35]. However, studies show that the activation of *NF-kB* and *MAPK* pathways results in the induction of inflammatory genes [36]. Aberrant *TNFAIP3* expression could also lead to inflammation and tissue damage [37]. Consistent with these studies, we found the number of neutrophils was higher in severe COVID-19 patients, and these patients had a higher expression of *NFKBIA* and *TNFAIP3* than the patients with mild or no infection [38].

Particularly, the P2Y_2_ receptor (P2Y_2_R), encoded by the *P2RY2* gene, is implicated in a wide range of inflammatory lung diseases whose pathogenesis overlaps with SARS-CoV-2 [39]. P2Y_2_R is activated by extracellular nucleotides ATP and UTP, which are released from cells upon injury or stress and play a major role in the initiation and maintenance of inflammation and immune modulation [39]. For instance, P2Y_2_R activation by ATP stimulates neutrophil recruitment into lungs, the release of neutrophil granular content, and directed migration of dendritic cells and eosinophils [40–43]. Besides, P2Y_2_R is expressed on pulmonary endothelial cells and its activation enhances *VCAM-1* expression facilitating leukocyte adhesion [44]. P2Y_2_R activation on airway epithelium mediates secretion of mucin and the proinflammatory cytokine *IL-33* [28, 45]. In addition to *IL-33*, P2Y_2_R mediates the production of several cytokines that are directly implicated in SARS-CoV-2 pathogenesis, including *IL-6, IL-1β, TNF-α, CXCL-10*, and *IFN-γ* [46–48]. Interestingly, *IFN-γ*, paralleled with P2Y_2_R, is strongly associated with our proposed signature. Furthermore, P2Y_2_R is known to cooperate with pannexin-1 (PANX-1) channel protein that mediates passive transport of ATP, which triggers lung inflammation and regulates the life cycle of multiple viruses through enhancing viral binding to host cells, uptake, and replication [49–53]. Hence, PANX-1 and probenecid (an FDA-approved PANX-1 inhibitor) have been recently suggested for further investigation in the efforts to develop a COVID-19 treatment [54]. Collectively, our signature correlations, consistent with a large body of literature, suggest a potential role for P2Y_2_R in the pathogenesis of SARS-CoV-2.

*IFIT* inhibits virus replication by binding, regulating the functions of cellular, viral proteins, and RNAs [55]. *IFIT2* possesses antiviral activity against the SARS-CoV-2 virus by acting on the capped viral mRNA and protects from lethal vesicular stomatitis virus neuropathogenesis [56]. Gene expression distribution in ridge plots of neutrophils, basal cells, dendritic cells, T cells, and macrophages show that *IFIT2* was expressed higher in severe patients than the healthy or mild patients. *IFIT2* expression is essential for an antiviral response [57]. Thus, *IFIT2* may have a function in the host immune response [57].

Furthermore, *CXCL2, CXCL,3,* and *CCL20* were found upregulated and identified in early infection models of SARS-CoV-2. Therefore, others proposed targeting these chemokine ligands as an effective therapeutic target during viral infection [22]. In our CMAP query, it also was found the knockdown of *CXCL2* has a robust negative connection (CS − 97.87), supporting this strategy. The *PPP1R15A was* also critical for the survival of infected cells and multiplication [52]. *PPP1R15A* expression was reported higher in cells with very high levels of SARS-CoV-2 RNA [53].

The signature developed in this study, from early transcriptional changes due to SARS-CoV-2 infection, was able to capture infection even in the postmortem lung biopsy samples accurately. Thus, the gene signature not only captures the putative gene expression but also provides a robust snapshot of the more persistent alterations in gene expression due to SARS-CoV-2 infection regardless of the duration of infection. Some of the genes in the signatures showed differential expression in various immune cells and may indicate the severity of the infection. For example, *TNFAIP3*, *PPP1R15A*, *NFKBIA*, and *IFIT2* have bimodal gene expression in the immune cells of severely infected patients compared to healthy or mildly infected patients, while the chemokine genes or chemokine inducible genes, *IL1A, CXCL2, CXCL3, CCL20,* and *PTX3* showed no variance. Overexpression of *TNFAIP3*, *PPP1R15A*, and *NFKBIA* genes induces proinflammatory cytokines and interferons. On the contrary, *UPC2* showed decreased expression in dendritic cells, macrophages, and natural killer cells from severe patients compared to healthy or mildly infected individuals. Low *UPC2* expression may indicate mitochondrial dysfunction, reactive oxygen species (ROS) accumulation, and more severe vascular disease [58, 59].

Cyclooxygenase (COX) inhibitors such as ibuprofen, celecoxib negatively regulate the *PI3K* pathway. It has been postulated that these inhibitors suppress *NF-kb* and *TNF-α* induces *JNK*, *MAPK,* and *ERK* activation via the *AKT* pathway, thus downregulating genes for inflammation and proliferation [60]. Ibuprofen is a common anti-inflammatory and antipyretic agent available over the counter. For COVID-19 related fever and pain control, recommendations on using nonsteroidal anti-inflammatory drugs (NSAIDs) such as ibuprofen in COVID-19 have been inconsistent since the beginning of the pandemic. French authorities initially recommended against using ibuprofen in COVID-19 patients due to a possible increased expression of the *ACE2* receptor and likely risk of increased viral entry to cause the infection. Later, this was disputed and several studies were recommended continuing ibuprofen [61]. However, in our CMAP query, ibuprofen had a strong negative connection in lung, colon and hepatic cancer cell lines (HCC515, HT29 and HEPG2, respectively) compared to a strong positive connection in a renal cancer cell line (HA1E) with the infection signature. Therefore, depending on cell types, ibuprofen may show different activities and the role of ibuprofen in COVID-19 treatment needs to be explored further.

Both MEK and HDAC inhibitors are used as anticancer drugs and modulate the immune response, induce cell cycle arrest, differentiation, and death. Additionally, HDAC inhibitors have been shown to repress *TMPRSS2-ERG* expression in prostate cancer [62, 63]. Therefore, HDAC inhibitors’ role in suppressing *TMPRSS2-ERG* may contribute to less efficient SARS-CoV-2 viral entry into the cells. These drugs are costly and have serious side effects. On the other hand, antidepressants are known for immunomodulatory effects, with several classes decreasing the production of proinflammatory cytokines and increasing the production of anti-inflammatory cytokines [64]. Maprotiline, a TCA, structurally different from other TCAs, had a strong negative connection in our analysis. Antidepressants such as TCAs and selective serotonin reuptake inhibitors have previously reported antiviral, immunomodulatory effects and antioxidant properties [65]. Although data are limited on the innate and adaptive immune effects of TCAs, they appear to have anti-inflammatory effects detected via *TNF-α* and *IL-6* [66].

There were several limitations to this study, including the scarcity of publicly available SARS-CoV-2 transcriptional, clinical, and drug response data preventing better characterize of the virus’s role in drug response. A limited number of only 12 samples of 24-hour post-infection were used to generate the signature. More diverse samples at various post-infection time points with additional replicates may improve the robustness of the signature. There was also limited signature data available through CMAP database which uses cancer cell lines for perturbation studies. Thus, the gene expression of human cells in vivo may be different than in these immortalized cell lines. Gene expression data from cell lines treated with newer drugs, such as remdesivir or other antivirals in clinical trials, are not available. This prevented further validation of the signature in newer or excluded drugs. Gene expression-based analysis using a single time point data provided a snapshot of the infection activity at that time point. The signature essentially captured the minimal gene set to define the infection status rather than early or late infection status. Although our signature was able to accurately predict infection status in patients with an unknown stage of SARS-CoV-2 infection, future in vitro studies with serial time points are required to better understand how the host response evolves due to infection with time.

In this study, the present work demonstrated that SARS-CoV-2 viral infection stimulates a unique response in host cells captured by using the 25-gene signature. Select genes in the signature may also indicate the severity of the infection in the host. Additionally, several potential drug targets were identified in the CMAP database. In all, the SARS-CoV-2 signature may help advance our understanding of both infection mechanisms and search for effective COVID-19 treatments.

## Conclusion

The 25-gene SARS-CoV-2 infection signature accurately predicted SARS-CoV-2 infection status in various lung samples, such as BALF cells, PBMCs, and postmortem lung biopsies in humans. Additionally, candidate SARS-CoV-2 therapies were identified with this signature. These signature genes may be utilized to determine the disease severity of COVID-19 in the infected patients’ BALF single-cell expression profiles.

## Methods

### Datasets

This study aimed to generate and validate the SARS-CoV-2 infection signature. The design and setting of the study by using multiple publicly available datasets were shown in Fig. S12. An RNA-Seq dataset from cell lines and patient samples were downloaded from the NCBI Gene Expression Omnibus (GEO) database (accession no. GSE147507) [26]. Human-derived cell lines 24-hour post-infection with SARS-CoV-2 and their associated controls were included for the signature generation and testing. Specifically, series 5, 6, 7, and 16 of cell lines A549, A549-ACE2 (*ACE2* overexpressed in A549 cell line), Calu-3, infected with SARS-CoV-2 and mock-treated were used as training sets, while series 2 and 15 were used as test sets [26]. Series 2 is A549 cell lines infected with low SARS-CoV-2 infection (MOI 0.2) [26]. The A549 cells are known to have low expression of the viral receptor *ACE2*. Therefore, A549 lung alveolar cells are relatively non-permissive to SARS-CoV-2 replication compared to Calu-3 cells, 0.1% versus 15% total reads, respectively. However, *ACE2* overexpressed in A549 cell lines were used for signature generation, and series 2 (A549 cell lines without *ACE2* overexpression) was used for internal validation. Series 15 contained samples from postmortem COVID19 patients and healthy lung biopsies (Table S3). This series was used to internally validate the signature in the patient samples.

Another independent validation dataset was downloaded from Genome Sequence Archive (GSA) in National Genomics Data Center, Beijing Institute of Genomics (BIG), Chinese Academy of Science (https://bigd.big.ac.cn, accession no. CRA002390). Four BALF samples from two patients with two replicates, PBMC samples from three infected patients and three healthy individuals were included in this dataset. Another dataset of BALF samples from the healthy control RNA-Seq dataset was obtained from the SRA database (SRAdb sample ids SRR10571724, SRR10571730, SRR10571732; Table S4) [67].

Finally, a scRNA-Seq dataset was used to comprehensively characterize the signature genes in single cells from BALF cells (GSE145926) [27]. scRNA-Seq data was generated using the 10X genomics platform from BALF cells of six severe/critical COVID-19 patients, three moderate COVID-19 patients and three healthy controls.

### Bioinformatics analysis

#### Ingenuity pathway analysis and RNA-Seq data processing

Raw read counts from GSE147507 were normalized by the DESeq2 median of ratios normalization method, followed by the differentially expressed gene analysis [68]. Genes with *p-adj* < 0.05 and *log*_*2*_*FoldChange* > 1 or < − 1 were considered as significantly differential expressed genes (DEGs). Ingenuity Pathway Analysis (IPA) was used to analyze the biological enrichment pathway of SARS-CoV-2 with DEGs [69]. *FastQC* was utilized to perform quality control for the raw fastq files of CRA002390, SRR10571724, SRR10571730, and SRR10571732 [70]. Sequencing reads were processed for library adapter removal and initial filtering by using *Trimmomatic* [71]. The *STAR* software package was used to align reads to a human reference genome (GRCH38) [72]. PCR replicates mapped in the human genome were removed with *picard MarkDuplicates* program (v2.22.7) [73]. Then, *featureCounts* was used to quantify the reads [74].

#### Batch adjustment

To minimize confounding batch effects between the different series of data, further data processing was performed. First, variances between the different cell line data were visualized using principal component analysis (PCA) [19, 75]. Significant batch effects were observed between all training and test RNA-Seq datasets. Using the *ComBat* function from the R package *sva* (v3.34.0), confounding batch effects were adjusted [76]. Within the GSE147507 dataset, the batch adjustment was performed considering each series separately since each series had different cell types with different MOIs. Following batch adjustment, a second PCA was performed to confirm the resolution of the batch effect. Series 5, 6, 7, and 16 were separated into two major groups — mock-treated and SARS-CoV-2 infected samples to generate signatures.

#### Signature generation and validation

To identify the minimum set of genes representing the status of the SARS-CoV-2 infection, cell line data were acquired from the NCBI GEO database (GSE147507). First, data were normalized by using the *DESeq2* median of ratio method, followed by batch adjustment using the *ComBat function* from the *sva R package* (Version 3.34.0). *Adaptive Signature Selection and InteGratioN* (ASSIGN; version 1.9.1) was utilized to generate the gene signature representative of SARS-CoV-2 infection [25]. ASSIGN is a semi-supervised pathway profiling toolkit that uses the Bayesian variable selection approach to different genes expressing a biological condition, such as SARS-CoV-2 infection for this study [25]. These genes were selected based on their signal strengths and weights, where the higher the value generated, the more significant contribution of the genes to the SARS-CoV-2 infection-related transcriptional activity [25].

With ASSIGN using the *assign.wrapper* function with default settings, gene signatures were generated by producing gene list lengths consisting of 25 genes ranging to 500 genes. The gene lists were produced in 25 gene increments, e.g., 25, 50, 75, 100, 125, and so on, up to 500 genes. SARS-CoV-2 infection activity was analyzed for each training sample using LOOCV. Predicted infection activity values generated ranged between zero to one, where “0” indicates no infection, and “1” indicates maximum infection activity. Series 5, 6, 7, and 16 were specified as the training datasets, while series 15 and 2 were test datasets. Series 2, A549 cell lines, consisted of control and very low SARS-CoV-2 infected samples, and series 15 dataset contained postmortem lung biopsy samples from patients with and without SARS-CoV-2 infection. While running each prediction in test and validation datasets, ASSIGN’s adaptive background feature was used to further correct the background transcriptional variation due to the cell line-specific and background gene expression variances. Finally, an independent external validation was performed in RNA-Seq datasets (CRA002390, SRR10571724, SRR10571730, and SRR10571732) from COVID-19 patients and healthy controls.

#### Characterization of signature genes in single cells

R package *Seurat* was used for data (GSE145926) normalization with *NormalizeData* function. Feature counts of each cell were divided by the total counts for that cell multiplied by a scaler factor (1e6), then natural-log transformed [77]. The normalized data were then integrated for batch effect adjustment and Uniform manifold approximation and projection (UMAP) clustering [77]. After a quality control check, *FindALLMarkers* was used to find cell markers for all clusters. Clusters were annotated based on canonical cell markers (Table S1). Different cell types were identified in severe/critical, moderate patients, and healthy control samples. Signature genes from the RNA-Seq data were evaluated in the scRNA-Seq dataset by using the *DoHeatmap* function with scaled expression values. *RidgePlot* was used to generate the distribution of signature genes’ expressions in various types of cells.

### Analysis of SARS-CoV-2 transcriptional activity for perturbagen detection

CSs were assessed with the signature gene list using a CMAP query to identify the most similar and dissimilar perturbagen signatures to our SARS-CoV-2 infection signature in the CMAP database with more than a million perturbation experiments [78]. The CMAP query finds similarities and dissimilarities across the curated expression profiles of various perturbations, including compounds, overexpressions, and knockdowns. CS is a quantitative score between a query gene-list and a perturbagen that ranges from − 100 (opposing signature) to 100 (same signature). CS of − 90 or lower for dissimilarity and 90 or higher for similarity were considered as strong connections.

## Supplementary Information

**Additional file 1: Fig. S1.** Leave-one-out-cross-validation scatter plot showing SARS-CoV-2 infection activity in the training cell line samples. **Fig. S2.** The key (top-scoring) 16 bio-functions in four series of data infected with SARS-CoV-2 were obtained through Ingenuity Pathway Analysis (IPA). **Fig. S3.** Uniform Manifold Approximation and Projection (UMAP) plot of cells from bronchoalveolar lavage fluid cells (*n*=12) show distinct clusters predominantly determined by cell type. **Fig. S4.** Expression of signature genes in basal cells from patient groups. **Fig. S5.** Expression of signature genes in dendritic cells from patient groups. **Fig. S6.** Expression of signature genes in macrophages from patient groups. **Fig. S7.** Expression of signature genes in naïve CD4+ T cells from patient groups. **Fig. S8.** Expression of signature genes in natural killer cells from patient groups. **Fig. S9.** Expression of signature genes in plasma cells from patient groups. **Fig. S10.** Expression of signature genes in T cells from patient groups. **Fig. S11.** Connectivity Scores (CSs) for Genetic perturbations with the 25-gene SARS-CoV-2 Infection Signature. **Fig. S12.** Data processing steps used in SARS-CoV-2 gene expression signature generation, testing and validation in various datasets. **Table S1.** Cell markers used to identify cell types in single-cell RNA-Sequencing dataset GSE145926. **Table S2.** Selected Connectivity Score (CS) with the 25-gene SARS-CoV-2 infection signature from the ConnectivityMap (CMAP) database. **Table S3.** Description of the samples from GSE147507 used for SARS-CoV-2 signature generation and internal validation. **Table S4.** Description of the external validation human datasets used for SARS-CoV-2 signature.

## Data Availability

The data used in the analyses described here are freely accessible. GSE147507, GSE145926,  SRR10571724, SRR10571730, SRR10571732 datasets are available in NCBI GEO, and CRA002390 is available in the BIG database. All RNA-Seq data analyses except the ConnectivityMap query and Ingenuity Pathway Analysis were performed in R version 3.6.1 and Bioconductor version 3.7 (R Core Team, 2014; http://www.R-project.org/). All codes are available at https://github.com/yueli8/COVID-19.

## Abbreviations

CDC: Centers for Disease Control and Prevention
ACE: angiotensin-converting enzyme
ARB: angiotensin receptor blocker
RNA-Seq: RNA Sequencing
COVID-19: 2019 coronavirus pandemic
SARS-CoV-2: Severe Acute Respiratory Syndrome Coronavirus 2
NP: Nasopharyngeal
OP: Oropharyngeal
RT-PCR: Reverse transcription polymerase chain reaction
BALF: Bronchoalveolar lavage fluid
ACE2: Angiotensin-converting enzyme 2
FDA: Food and Drug Administration
RECOVERY: Randomized Evaluation of COVid-19 thERapY
PBMC: Peripheral blood mononuclear cell
CMAP: ConnectivityMap
ASSIGN: Adaptive Signature Selection and InteGratioN
LOOCV: Leave-one-out-cross-validation
IPA: Ingenuity Pathway Analysis
MOI: Multiplicity of Infection
NK: Natural killer
TCA: Tricyclic antidepressants
RAS: Renin-angiotensin system
ACE: Angiotensin-converting enzyme
BK: Bradykinin
GPCR: G-protein coupled receptors
P2Y_2_R: P2Y_2_ receptor
PANX-1: Pannexin-1
ROS: Reactive oxygen species
COX: Cyclooxygenase
NSAIDs: Nonsteroidal anti-inflammatory drugs
GEO: Gene Expression Omnibus
GSA: Genome Sequence Archive
BIG: Beijing Institute of Genomics
scRNA-Seq: Single-cell RNA-Sequencing
DEGs: Differentially expressed genes
UMAP: Uniform Manifold Approximation and Projection
CSs: Connectivity scores

## References

[1] Cavalli E, Petralia M, Basile M, Bramanti A, Bramanti P, Nicoletti F (2020). Transcriptomic analysis of COVID-19 lungs and bronchoalveolar lavage fluid samples reveals predominant B cell activation responses to infection. Int J Mol Med.

[2] Patel MR, Carroll D, Ussery E, Whitham H, Elkins CA, Noble-Wang J (2020). Performance of oropharyngeal swab testing compared to nasopharyngeal swab testing for diagnosis of COVID-19 —United States, January–February 2020. Clin Infect Dis.

[3] Tang Y, Schmitz JE, Persing DH, Stratton CW (2020). Laboratory diagnosis of COVID-19: current issues and challenges. J Clin Microbiol.

[4] Wang W, Xu Y, Lu R (2020). Detection of SARS - CoV - 2 in Different Types of Clinical Specimens. JAMA.

[5] Li Q, Guan X, Wu P, Wang X, Zhou L, Tong Y (2020). Early transmission dynamics in Wuhan, China, of novel coronavirus-infected pneumonia. N Engl J Med.

[6] Murthy S, Gomersall CD, Fowler RA (2020). Care for Critically ill Patients with COVID-19. JAMA.

[7] Hou Y, Zhao J, Martin W, Kallianpur A, Chung MK, Jehi L (2020). New insights into genetic susceptibility of COVID-19: an ACE2 and TMPRSS2 polymorphism analysis. BMC Med.

[8] Thomson G (2020). COVID-19: Social distancing, ACE 2 receptors, protease inhibitors and beyond?.

[9] Hoffmann M, Kleine-Weber H, Schroeder S, Krüger N, Herrler T, Erichsen S (2020). SARS-CoV-2 Cell Entry Depends on ACE2 and TMPRSS2 and Is Blocked by a Clinically Proven Protease Inhibitor. Cell.

[10] Wu C, Zheng M (2019). Single-cell RNA expression profiling shows that ACE2 , the putative receptor of COVID-2019, has significant expression in nasal and mouth tissue , and is co-expressed with TMPRSS2 and not co-expressed with SLC6A19 in the tissues.

[11] Zipeto D, Palmeira J (2020). ACE2 / ADAM17 / TMPRSS2 interplay may be the main risk factor for COVID-19.

[12] Palau V, Riera M, Soler MJ (2020). ADAM17 inhibition may exert a protective effect on COVID-19. Nephrol Dial Transplant.

[13] Mehta P, McAuley DF, Brown M, Sanchez E, Tattersall RS, Manson JJ (2020). COVID-19: consider cytokine storm syndromes and immunosuppression. Lancet.

[14] Lippi G, Simundic AM, Plebani M (2020). Potential preanalytical and analytical vulnerabilities in the laboratory diagnosis of coronavirus disease 2019 (COVID-19). Clin Chem Lab Med.

[15] Sanders JM, Monogue ML, Jodlowski TZ, Cutrell JB (2020). Pharmacologic treatments for coronavirus disease 2019 (COVID-19): a review. JAMA.

[16] Wang Y, Zhang D, Du G, Du R, Zhao J, Jin Y (2020). Remdesivir in adults with severe COVID-19: a randomised, double-blind, placebo-controlled, multicentre trial. Lancet.

[17] RECOVERY Collaborative Group. Dexamethasone in Hospitalized Patients with Covid-19 — Preliminary Report. N Engl J Med. 2020:1–11. 10.1056/NEJMoa2021436.

[18] Bild AH, Yao G, Chang JT, Wang Q, Potti A, Chasse D (2006). Oncogenic pathway signatures in human cancers as a guide to targeted therapies. Nature.

[19] Rahman M, Macneil SM, Jenkins DF, Shrestha G, Wyatt SR, Mcquerry JA (2017). Activity of distinct growth factor receptor network components in breast tumors uncovers two biologically relevant subtypes.

[20] Nikolayeva I, Bost P, Casademont I, Duong V, Koeth F, Prot M (2018). A blood RNA signature detecting severe disease in young dengue patients at hospital arrival. J Infect Dis.

[21] Babayan SA, Orton RJ, Streicker DG (2018). Predicting reservoir hosts and arthropod vectors from evolutionary signatures in RNA virus genomes. Science.

[22] Xiong Y, Liu Y, Cao L, Wang D, Guo M, Jiang A (2020). Transcriptomic characteristics of bronchoalveolar lavage fluid and peripheral blood mononuclear cells in COVID-19 patients. Emerg Microbes Infect.

[23] Itadani H, Mizuarai S, Kotani H (2008). Can systems biology understand pathway activation? Gene expression signatures as surrogate markers for understanding the complexity of pathway activation. Curr Genomics.

[24] Liu J, Campen A, Huang S, Peng S-B, Ye X, Palakal M (2008). Identification of a gene signature in cell cycle pathway for breast cancer prognosis using gene expression profiling data. BMC Med Genet.

[25] Shen Y, Rahman M, Piccolo SR, Gusenleitner D, El-chaar NN, Cheng L (2015). ASSIGN : context-specific genomic profiling of multiple heterogeneous biological pathways. Bioinformatics.

[26] Blanco-Melo D, Nilsson-Payant BE, Liu WC, Uhl S, Hoagland D, Møller R (2020). Imbalanced Host Response to SARS-CoV-2 Drives Development of COVID-19. Cell.

[27] Liao M, Liu Y, Yuan J, Wen Y, Xu G, Zhao J (2020). Single-cell landscape of bronchoalveolar immune cells in patients with COVID-19. Nat Med.

[28] Kemp PA, Sugar RA, Jackson AD (2004). Nucleotide-mediated mucin secretion from differentiated human bronchial epithelial cells. Am J Respir Cell Mol Biol.

[29] Buszko M, Park JH, Verthelyi D, Sen R, Young HA, Rosenberg AS (2020). The dynamic changes in cytokine responses in COVID-19: a snapshot of the current state of knowledge. Nat Immunol.

[30] Burch RM (2013). Bradykinin receptors. Encycl Biol Chem Second Ed.

[31] Massrieh W, Derjuga A, Doualla-Bell F, Ku CY, Sanborn BM, Blank V (2006). Regulation of the MAFF transcription factor by proinflammatory cytokines in myometrial cells. Biol Reprod.

[32] Zhang Y, Cardell LO, Edvinsson L, Xu CB (2013). MAPK/NF-κB-dependent upregulation of kinin receptors mediates airway hyperreactivity: a new perspective for the treatment. Pharmacol Res.

[33] Cardoso AM (2020). COVID-19 and purinergic signaling: the need for investigation. Purinergic Signal.

[34] Liu T, Zhang L, Joo D, Sun SC (2017). NF-κB signaling in inflammation. Signal Transduct Target Ther.

[35] Mahmudpour M, Roozbeh J, Keshavarz M, Farrokhi S, Nabipour I (2020). COVID-19 cytokine storm: The anger of inflammation. Cytokine.

[36] Joung HJ, Jetten AM (2008). NF-κB-dependent transcriptional activation in lung carcinoma cells by farnesol involves p65/RelA (Ser276) phosphorylation via the MEK-MSK1 signaling pathway. J Biol Chem.

[37] Vereecke L, Beyaert R, van Loo G (2009). The ubiquitin-editing enzyme A20 (TNFAIP3) is a central regulator of immunopathology. Trends Immunol.

[38] Meizlish ML, Pine AB, Bishai JD, Goshua G, Nadelmann ER, Simonov M, et al. A neutrophil activation signature predicts critical illness and mortality in COVID-19. medRxiv. 2020. 10.1101/2020.09.01.20183897. 10.1182/bloodadvances.2020003568 PMC7908851 33635335

[39] Müller T, Idzko M (2012). P2Y receptors in lung inflammation. Wiley Interdiscip Rev Membr Transp Signal.

[40] Cicko S, Lucattelli M, Müller T, Lommatzsch M, De Cunto G, Cardini S (2010). Purinergic receptor inhibition prevents the development of smoke-induced lung injury and emphysema. J Immunol.

[41] Meshki J, Tuluc F, Bredetean O, Ding Z, Kunapuli SP (2004). Molecular mechanism of nucleotide-induced primary granule release in human neutrophils: Role for the P2Y2 receptor. Am J Physiol Cell Physiol.

[42] Idzko M, Dichmann S, Ferrari D, Di Virgilio F, La Sala A, Girolomoni G (2002). Nucleotides induce chemotaxis and actin polymerization in immature but not mature human dendritic cells via activation of pertussis toxin-sensitive P2y receptors. Blood.

[43] Idzko M, Dichmann S, Panther E, Ferrari D, Herouy Y, Virchow C (2001). Functional characterization of P2Y and P2X receptors in human eosinophils. J Cell Physiol.

[44] Vanderstocken G, Bondue B, Horckmans M, Di Pietrantonio L, Robaye B, Boeynaems J-M (2010). P2Y2 receptor regulates VCAM-1 membrane and soluble forms and eosinophil accumulation during lung inflammation. J Immunol.

[45] Kouzaki H, Iijima K, Kobayashi T, O’Grady SM, Kita H (2011). The danger signal, extracellular ATP, is a sensor for an airborne allergen and triggers IL-33 release and innate Th2-type responses. J Immunol.

[46] Douillet CD, Robinson WP, Milano PM, Boucher RC, Rich PB (2006). Nucleotides induce IL-6 release from human airway epithelia via P2Y 2 and p38 MAPK-dependent pathways. Am J Physiol Lung Cell Mol Physiol.

[47] Relvas LJM, Makhoul M, Dewispelaere R, Caspers L, Communi D, Boeynaems JM (2015). P2Y2R deficiency attenuates experimental autoimmune uveitis development. PLoS One.

[48] Salem M, Tremblay A, Pelletier J, Robaye B, Sévigny J (2018). P2Y6 receptors regulate CXCL10 expression and secretion in mouse intestinal epithelial cells. Front Pharmacol.

[49] Thorstenberg ML, Ferreira MVR, Amorim N, Canetti C, Morrone FB, Filho JCA (2018). Purinergic cooperation between P2Y2 and P2X7 receptors promote cutaneous leishmaniasis control: Involvement of pannexin-1 and leukotrienes. Front Immunol.

[50] Graziano F, Desdouits M, Garzetti L, Podini P, Alfano M, Rubartelli A (2015). Extracellular ATP induces the rapid release of HIV-1 from virus containing compartments of human macrophages. Proc Natl Acad Sci U S A.

[51] Krick S, Wang J, St-Pierre M, Gonzalez C, Dahl G, Salathe M (2016). Dual oxidase 2 (Duox2) regulates Pannexin 1-mediated ATP release in primary human airway epithelial cells via changes in intracellular pH and not H2O2 production. J Biol Chem.

[52] Lee BH, Hwang DM, Palaniyar N, Grinstein S, Philpott DJ, Hu J (2012). Activation of P2X7 receptor by ATP plays an important role in regulating inflammatory responses during acute viral infection. PLoS One.

[53] Zhang C, He H, Wang L, Zhang N, Huang H, Xiong Q (2017). Virus-triggered ATP release limits viral replication through facilitating IFN-β production in a P2X7-dependent manner. J Immunol.

[54] Swayne LA, Johnstone SR, Ng CS, Sanchez-Arias JC, Good ME, Penuela S (2020). Consideration of pannexin 1 channels in covid-19 pathology and treatment. Am J Physiol Lung Cell Mol Physiol.

[55] Fensterl V, Sen GC (2015). Interferon-induced Ifit proteins: their role in viral pathogenesis. J Virol.

[56] Siegfried A, Berchtold S, Manncke B, Deuschle E, Reber J, Ott T (2013). IFIT2 is an effector protein of type I IFN–mediated amplification of lipopolysaccharide (LPS)-induced TNF-α secretion and LPS-induced endotoxin shock. J Immunol.

[57] Tran V, Ledwith MP, Thamamongood T, Higgins CA, Tripathi S, Chang MW (2020). Influenza virus repurposes the antiviral protein IFIT2 to promote translation of viral mRNAs. Nat Microbiol.

[58] Pierelli G, Stanzione R, Forte M, Migliarino S, Perelli M, Volpe M (2017). Uncoupling protein 2: a key player and a potential therapeutic target in vascular diseases. Oxidative Med Cell Longev.

[59] Moriyama M, Chen I-Y, Kawaguchi A, Koshiba T, Nagata K, Takeyama H (2016). The RNA- and TRIM25-binding domains of influenza virus NS1. J Virol.

[60] Shishodia S, Koul D, Aggarwal BB (2004). Cyclooxygenase (COX)-2 inhibitor Celecoxib abrogates TNF-induced NF-κB activation through inhibition of activation of IκBα kinase and Akt in human non-small cell lung carcinoma: correlation with suppression of COX-2 synthesis. J Immunol.

[61] Moore N, Carleton B, Blin P, Bosco-Levy P, Droz C (2020). Does Ibuprofen Worsen COVID-19?. Drug Saf.

[62] Fortson WS, Kayarthodi S, Fujimura Y, Xu H, Matthews R, Grizzle WE (2011). Histone deacetylase inhibitors, valproic acid and trichostatin-a induce apoptosis and affect acetylation status of p53 in ERG-positive prostate cancer cells. Int J Oncol.

[63] Mollica V, Rizzo A, Massari F (2020). The pivotal role of TMPRSS2 in coronavirus disease 2019 and prostate cancer. Future Oncol..

[64] Kenis G, Maes M (2002). Effects of antidepressants on the production of cytokines. Int J Neuropsychopharmacol.

[65] Hamed MGM, Hagag RS (2020). The possible immunoregulatory and anti-inflammatory effects of selective serotonin reuptake inhibitors in coronavirus disease patients. Med Hypotheses.

[66] Eyre HA, Lavretsky H, Kartika J, Qassim A, Baune BT (2016). Modulatory effects of antidepressant classes on the innate and adaptive immune system in depression. Pharmacopsychiatry.

[67] Michalovich D, Rodriguez-Perez N, Smolinska S, Pirozynski M, Mayhew D, Uddin S, et al. Obesity and disease severity magnify disturbed microbiome-immune interactions in asthma patients. Nat Commun. 2019;10(1). 10.1038/s41467-019-13751-9. 10.1038/s41467-019-13751-9 PMC6911092 31836714

[68] Love MI, Huber W, Anders S (2014). Moderated estimation of fold change and dispersion for RNA-seq data with DESeq2.

[69] Jiménez-Marín Á, Collado-Romero M, Ramirez-Boo M, Arce C, Garrido JJ (2009). Biological pathway analysis by ArrayUnlock and ingenuity pathway analysis. BMC Proc.

[70] Conesa A, Madrigal P, Tarazona S, Gomez-Cabrero D, Cervera A, McPherson A (2016). A survey of best practices for RNA-seq data analysis. Genome Biol.

[71] Bolger AM, Lohse M, Usadel B (2014). Trimmomatic: a flexible trimmer for Illumina sequence data. Bioinformatics.

[72] Dobin A, Gingeras TR, Spring C, Flores R, Sampson J, Knight R (2016). Mapping RNA-seq with STAR. Curr Protoc Bioinform.

[73] Ebbert MTW, Wadsworth ME, Staley LA, Hoyt KL, Pickett B, Miller J, et al. Evaluating the necessity of PCR duplicate removal from next-generation sequencing data and a comparison of approaches. BMC Bioinformatics. 2016;17(Suppl 7). 10.1186/s12859-016-1097-3. 10.1186/s12859-016-1097-3 PMC4965708 27454357

[74] Liao Y, Smyth GK, Shi W (2014). feature Counts: an efficient general purpose program for assigning sequence reads to genomic features. Bioinformatics.

[75] Leek JT, Storey JD (2007). Capturing heterogeneity in gene expression studies by surrogate variable analysis. PLoS Genet.

[76] Leek JT, Johnson WE, Parker HS, Jaffe AE, Storey JD (2012). The SVA package for removing batch effects and other unwanted variation in high-throughput experiments. Bioinformatics.

[77] Stuart T, Butler A, Hoffman P, Hafemeister C, Papalexi E, Mauck WM (2019). Comprehensive integration of single-cell data. Cell.

[78] Subramanian A, Narayan R, Corsello SM, Peck DD, Natoli TE, Lu X (2017). A Next Generation Connectivity Map: L1000 Platform and the First 1,000,000 Profiles. Cell.

