# Supplementary material for: SARS-CoV-2 early infection signature identified potential key infection mechanisms and drug targets
Source: BMC Genomics. 2021 Feb 18;22:125. doi: 10.1186/s12864-021-07433-4 (PMC7889713; doi:10.1186/s12864-021-07433-4)
Supplement: Supplementary file 1 — Additional file 1: Fig. S1. Leave-one-out-cross-validation scatter plot showing SARS-CoV-2 infection activity in the training cell line samples. Fig. S2. The key (top-scoring) 16 bio-functions in four series of data infected with SARS-CoV-2 were obtained through Ingenuity Pathway Analysis (IPA). Fig. S3. Uniform Manifold Approximation and Projection (UMAP) plot of cells from bronchoalveolar lavage fluid cells (n=12) show distinct clusters predominantly determined by cell type. Fig. S4. Expression of signature genes in basal cells from patient groups. Fig. S5. Expression of signature genes in dendritic cells from patient groups. Fig. S6. Expression of signature genes in macrophages from patient groups. Fig. S7. Expression of signature genes in naïve CD4+ T cells from patient groups. Fig. S8. Expression of signature genes in natural killer cells from patient groups. Fig. S9. Expression of signature genes in plasma cells from patient groups. Fig. S10. Expression of signature genes in T cells from patient groups. Fig. S11. Connectivity Scores (CSs) for Genetic perturbations with the 25-gene SARS-CoV-2 Infection Signature. Fig. S12. Data processing steps used in SARS-CoV-2 gene expression signature generation, testing and validation in various datasets. Table S1. Cell markers used to identify cell types in single-cell RNA-Sequencing dataset GSE145926. Table S2. Selected Connectivity Score (CS) with the 25-gene SARS-CoV-2 infection signature from the ConnectivityMap (CMAP) database. Table S3. Description of the samples from GSE147507 used for SARS-CoV-2 signature generation and internal validation. Table S4. Description of the external validation human datasets used for SARS-CoV-2 signature. [file 12864_2021_7433_MOESM1_ESM.docx]

**Supplementary Information**

**SARS-CoV-2 early infection signature identified potential key infection mechanisms and drug targets**

Yue Li^1^, Ashley Duche^1^, Michael R. Sayer^1^, Don Roosan^2^, Farid G. Khalafalla^3^, Rennolds S. Ostrom^1^, Jennifer Totonchy^1^, Moom R. Roosan^1^

^1^Chapman University School of Pharmacy, Irvine, CA 92618

^2^ Western University of Health Sciences, College of Pharmacy, Pomona, CA 91766

^3^California Health Sciences University, College of Pharmacy, Clovis, CA 93612

**Table of Contents**

Fig. S1. Leave-one-out-cross-validation scatter plot showing SARS-CoV-2 infection activity in the training cell line samples………………………………………………………...................3

Fig. S2. The key (top-scoring) 16 bio-functions in four series of data infected with SARS-CoV-2 were obtained through Ingenuity Pathway Analysis (IPA). ……………………………...........4

Fig. S3. Uniform Manifold Approximation and Projection (UMAP) plot of cells from bronchoalveolar lavage fluid cells (n=12) show distinct clusters predominantly determined by cell type. ………………………………………………………………………………………..5

Fig. S4. Expression of signature genes in basal cells from patient groups………………..........6

Fig. S5. Expression of signature genes in dendritic cells from patient groups…………………7

Fig. S6. Expression of signature genes in macrophages from patient groups………..................8

Fig. S7. Expression of signature genes in naïve CD4+ T cells from patient groups…………....9

Fig. S8. Expression of signature genes in natural killer cells from patient groups………………………………………………………………………………………….10

Fig. S9. Expression of signature genes in plasma cells from patient groups. …………...........11

Fig. S10. Expression of signature genes in T cells from patient groups. ………………..........12

Fig. S11. Connectivity Scores (CSs) for Genetic perturbations with the 25-gene SARS-CoV-2 Infection Signature…………………………………………………………………….............13

Fig. S12. Data processing steps used in SARS-CoV-2 gene expression signature generation, testing and validation in various datasets……………………………………….......................14

Table S1: Cell markers used to identify cell types in single-cell RNA-Sequencing dataset GSE145926…………………………………………………………………………………….15

Table S2. Selected Connectivity Score (CS) with the 25-gene SARS-CoV-2 infection signature from the ConnectivityMap (CMAP) database…………………………………………………16

Table S3: Description of the samples from GSE147507 used for SARS-CoV-2 signature generation and internal validation………………………………………………………………34

Table S4: Description of the external validation human datasets used for SARS-CoV-2 signature…………………………………………………………………………………………35

References ………………………………………………………………………………………36


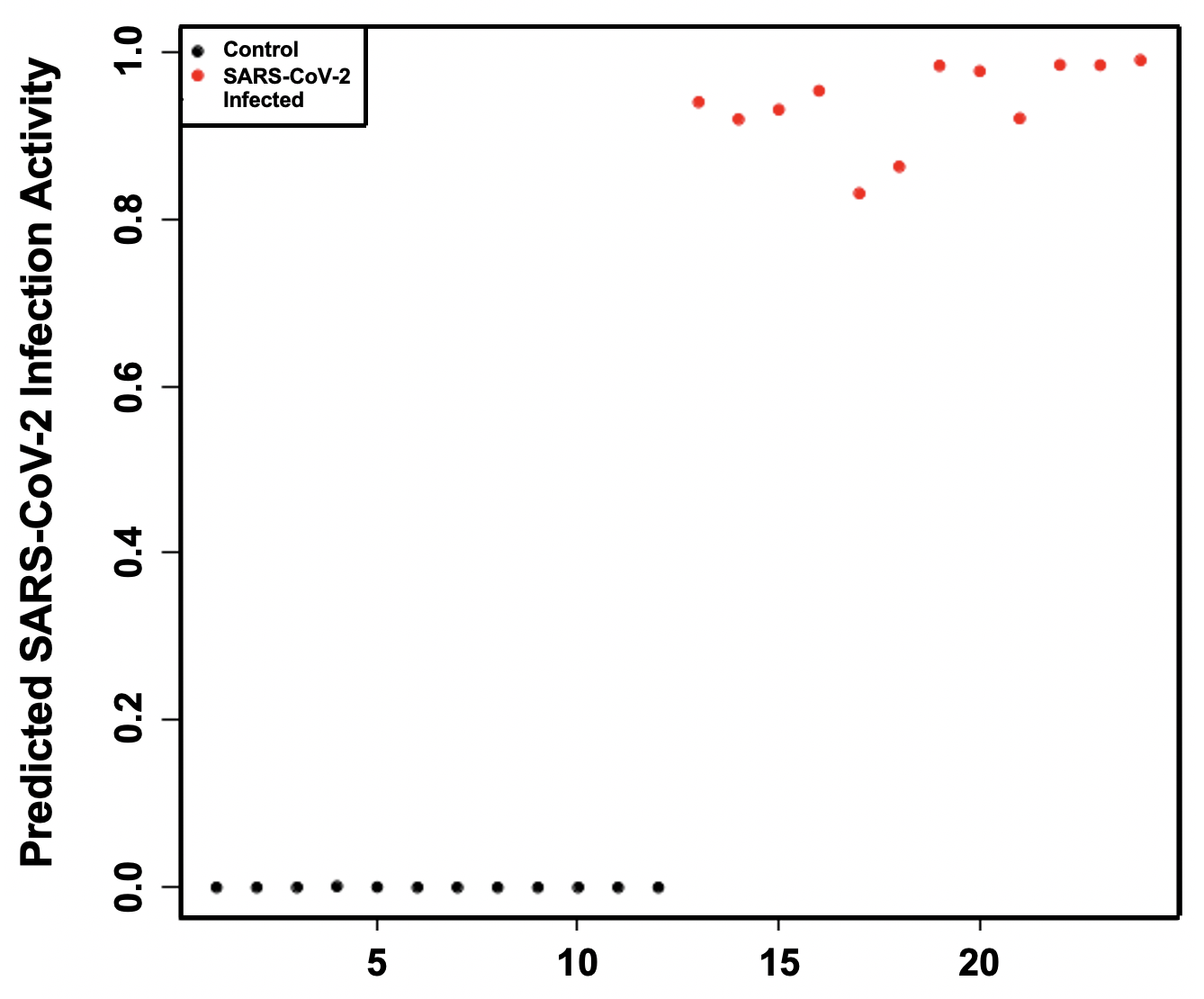


**Fig. S1. Leave-one-out-cross-validation scatter plot showing SARS-CoV-2 infection activity in the training cell line samples.** Values of zero represent no infection activity and one represents high infection activity.


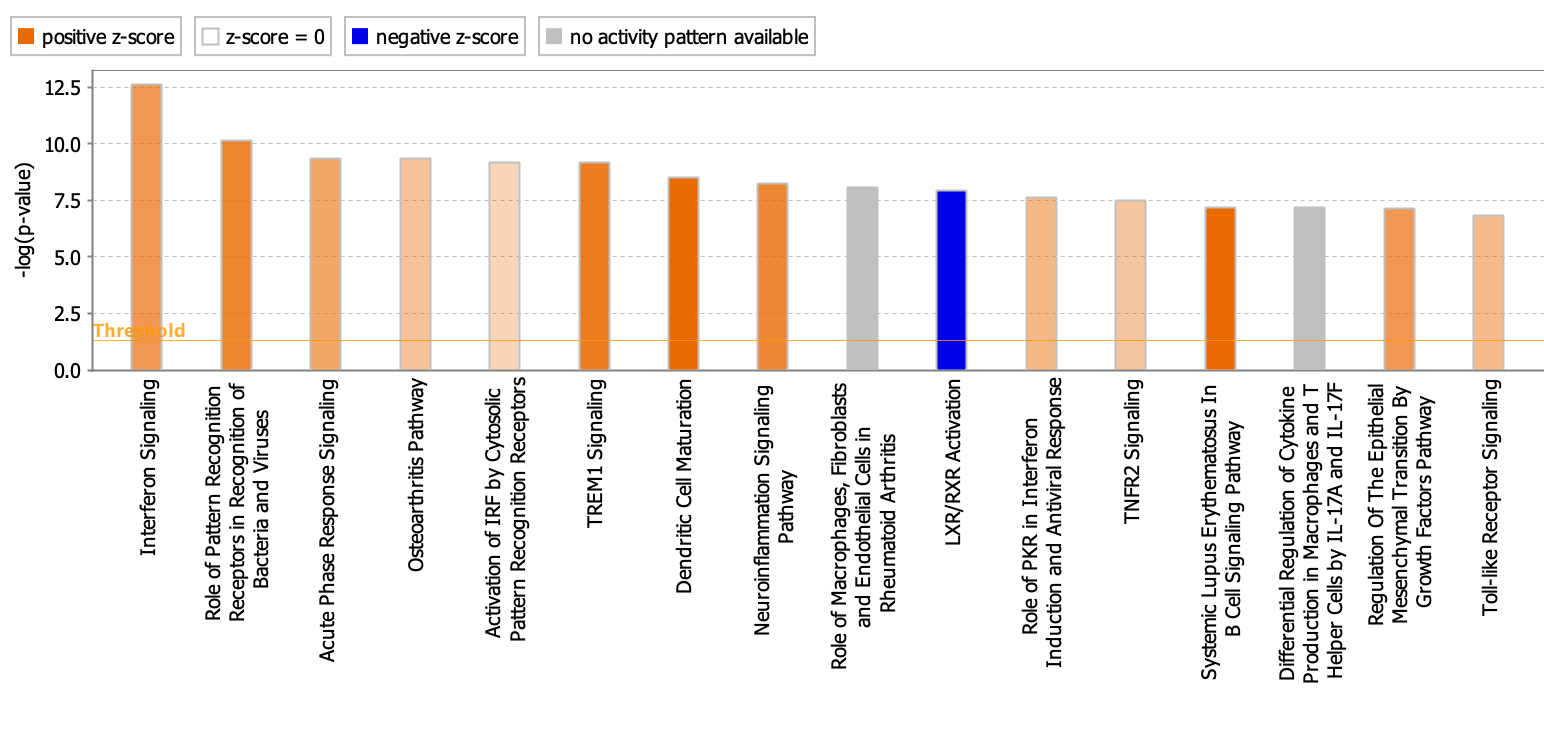


**Fig. S2. The key (top-scoring) 16 bio-functions in four series of data infected with SARS-CoV-2 were obtained through Ingenuity Pathway Analysis (IPA).** The most statistically significant bio-functions that were identified in the IPA infected analysis are listed here according to their *P-value* (-log). The threshold line corresponds to a *P-value* of 0.05.


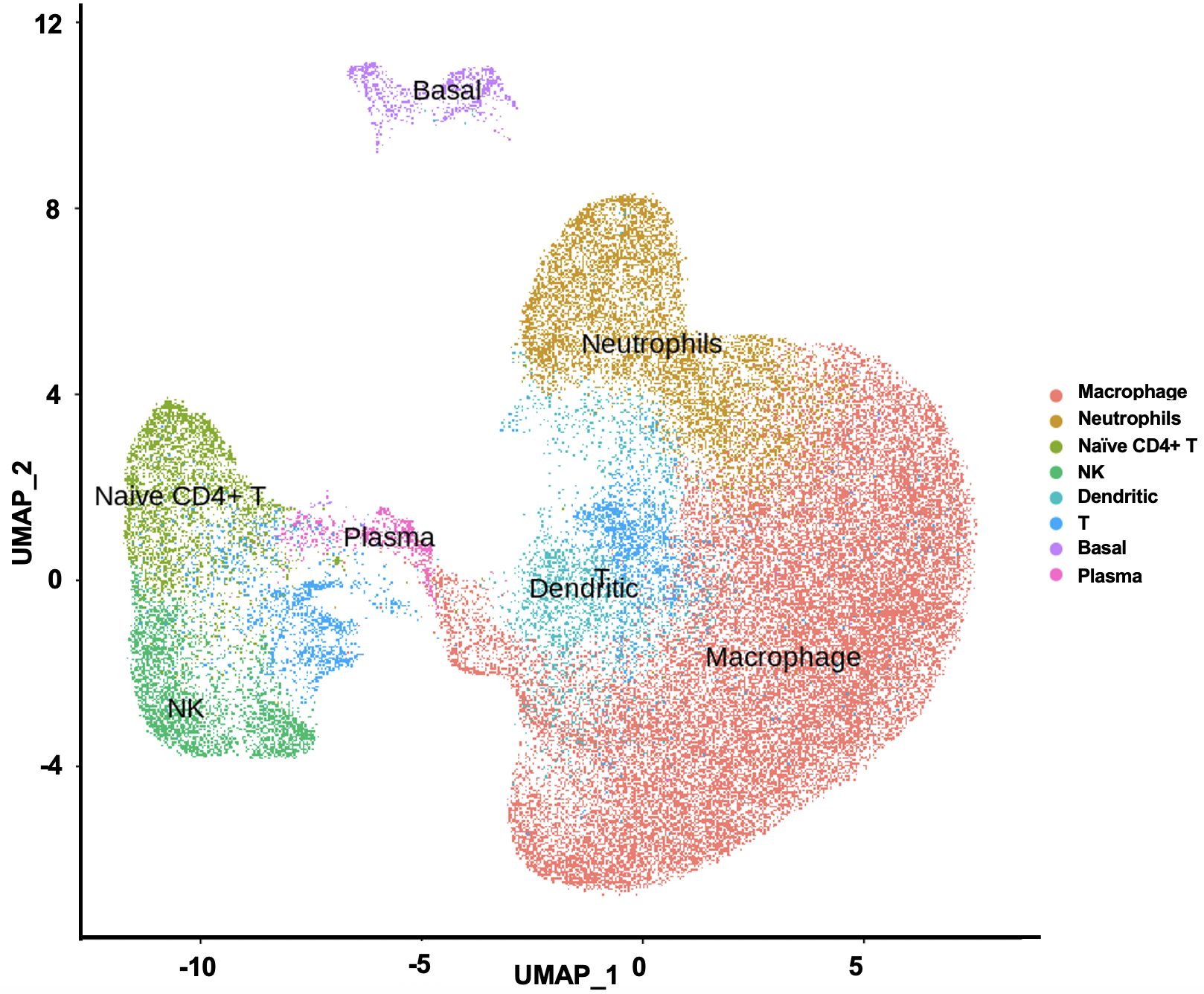


**Fig. S3. Uniform Manifold Approximation and Projection (UMAP) plot of cells from bronchoalveolar lavage fluid cells (n=12) show distinct clusters predominantly determined by cell type.** Each dot corresponds to a single cell and is colored according to cell type.


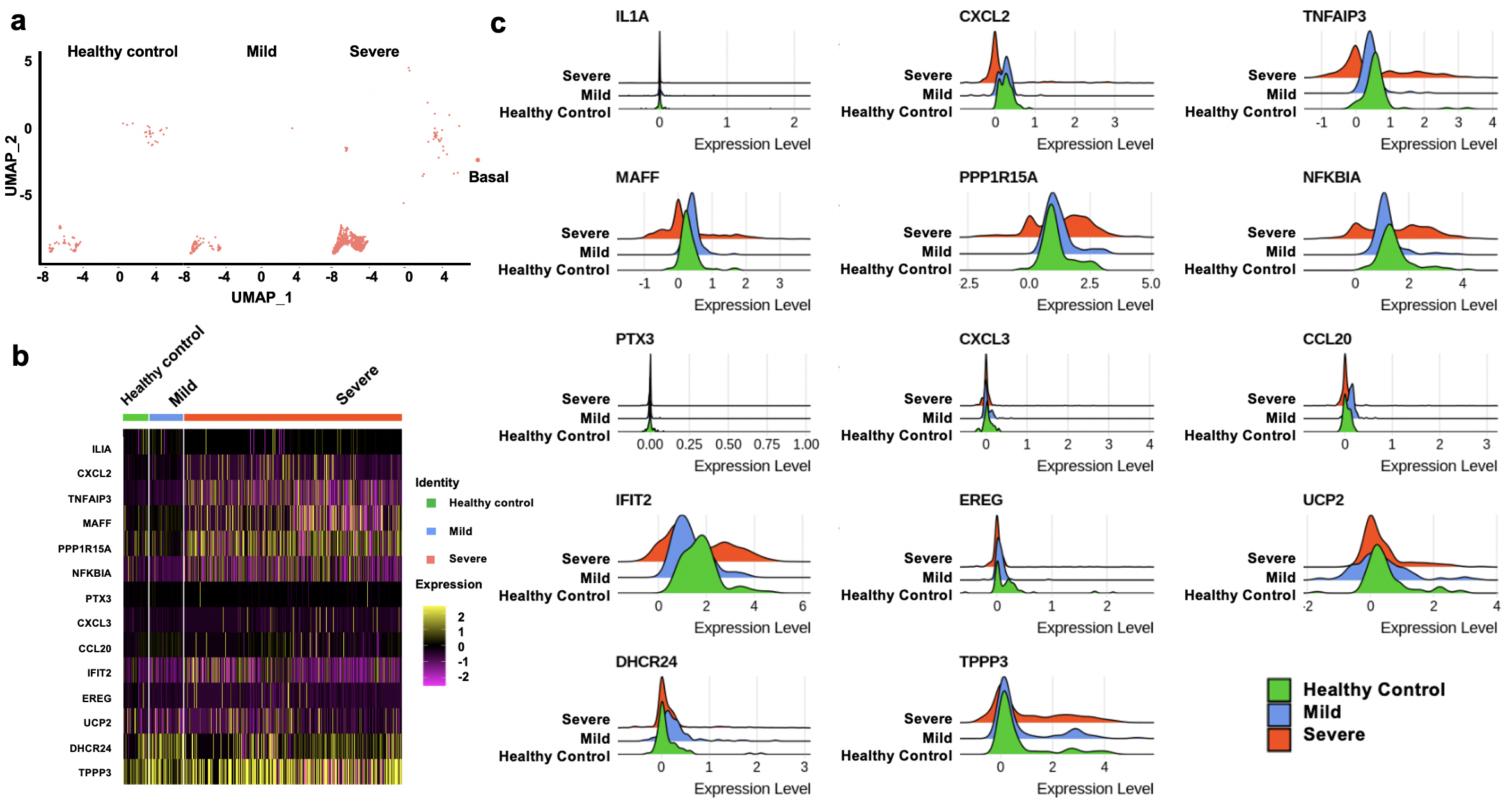


**Fig. S4.** **Expression of signature genes in basal cells from patient groups. a,** UMAP plots of the basal cells. Each dot corresponds to one single cell. **b,** Heatmap of fourteen signature genes in three groups. Each vertical bar represents a single cell. Column (cell identity) width is proportional to the number of cells present in that cluster. **c,** Distributions of signature gene expression shown in ridge plots. Red, blue and green colors represent gene expression values in severe, mild patients and healthy controls.


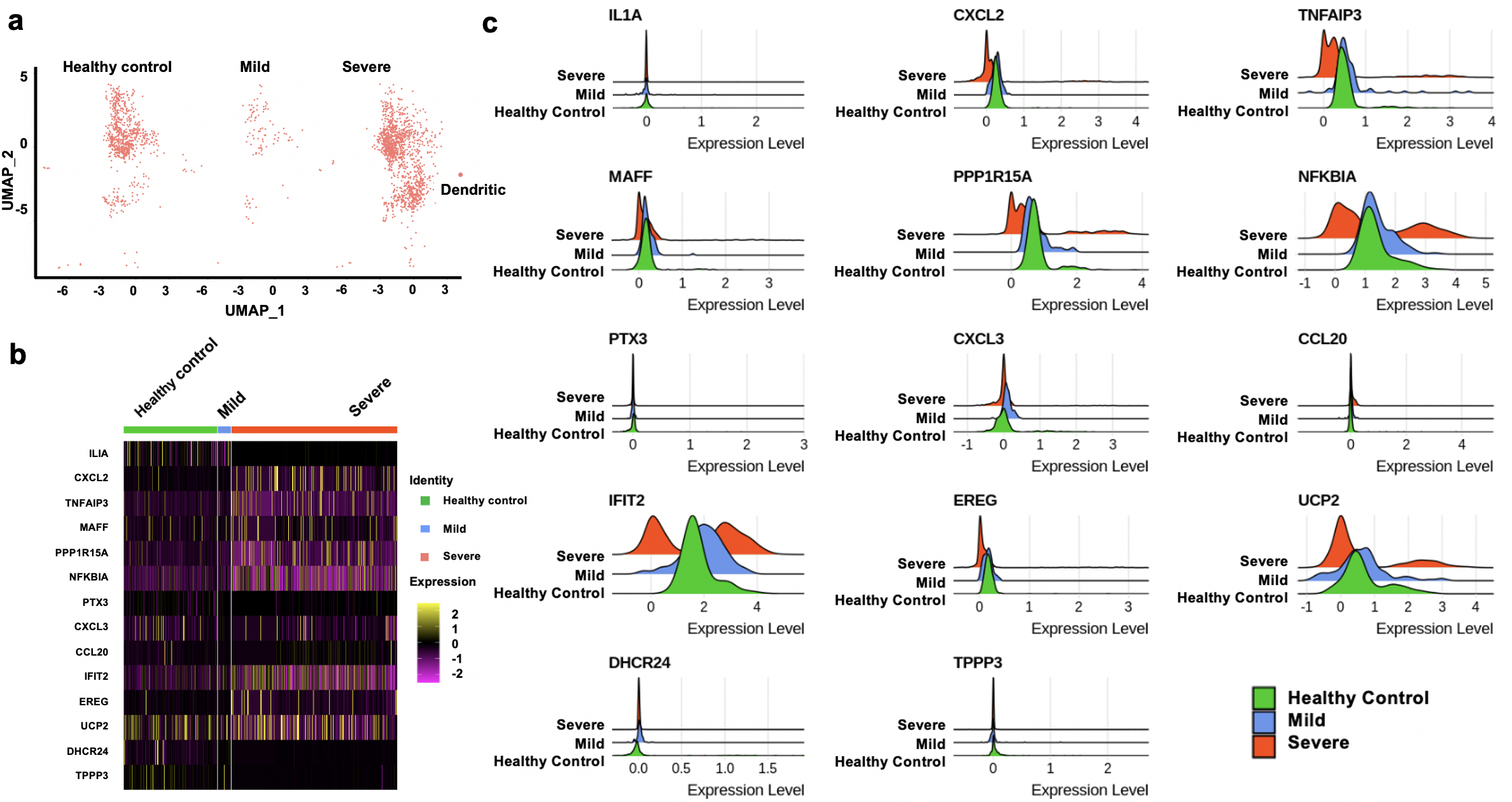


**Fig. S5.** **Expression of signature genes in dendritic cells from patient groups.** **a,** UMAP plots of the dendritic cells. Each dot corresponds to one single cell. **b,** Heatmap of fourteen signature genes in three groups. Each vertical bar represents a single cell. Column (cell identity) width is proportional to the number of cells present in that cluster. **c,** Distributions of signature gene expression shown in ridge plots. Red, blue and green colors represent gene expression values in severe, mild patients and healthy controls.


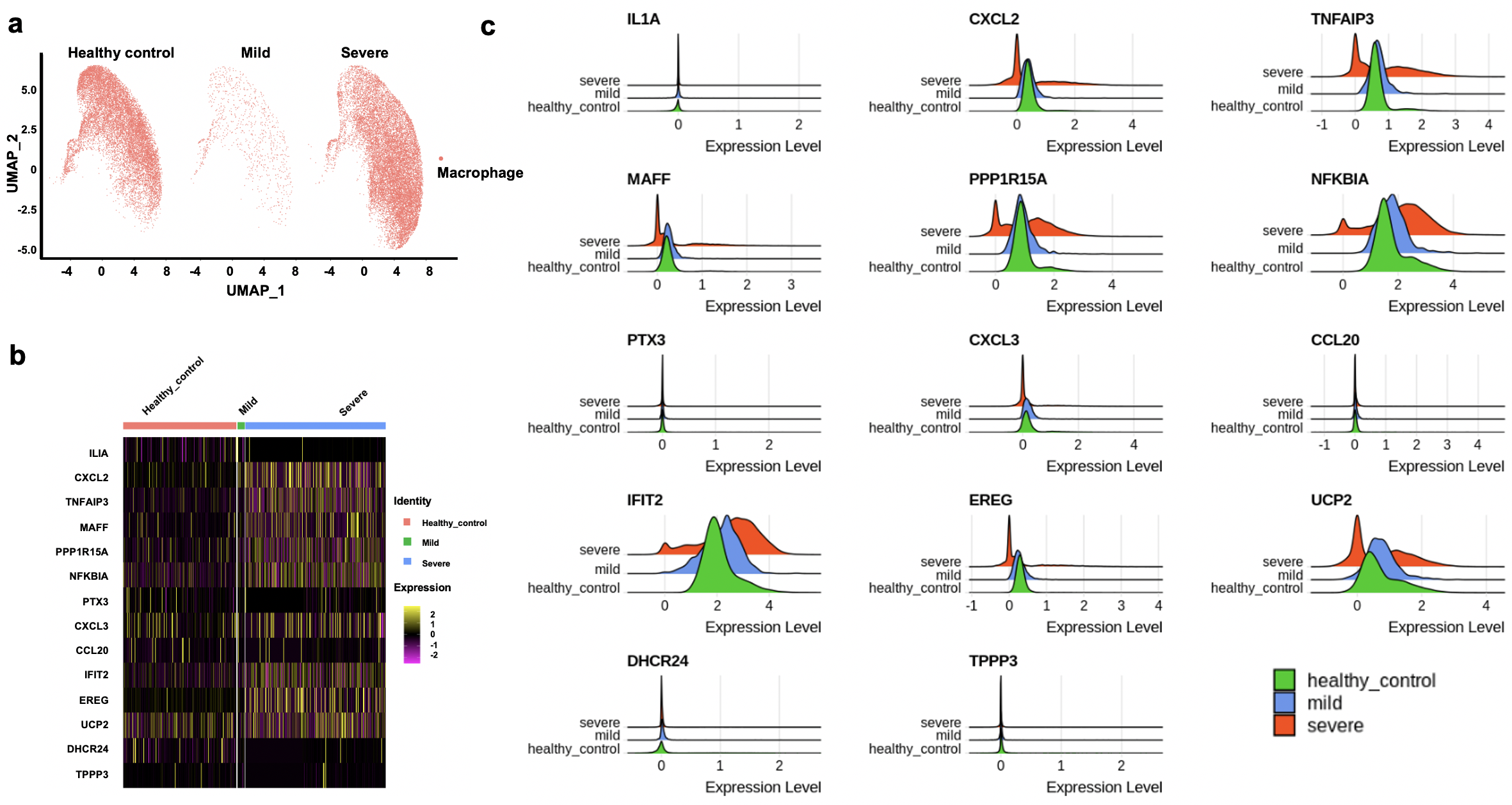


**Fig. S6. Expression of signature genes in macrophages from patient groups. a,** UMAP plots of the macrophage cells. Each dot corresponds to one single cell. **b,** Heatmap of fourteen signature genes in three groups. Each vertical bar represents a single cell. Column (cell identity) width is proportional to the number of cells present in that cluster. **c,** Distributions of signature gene expression shown in ridge plots. Red, blue and green colors represent gene expression values in severe, mild patients and healthy controls.


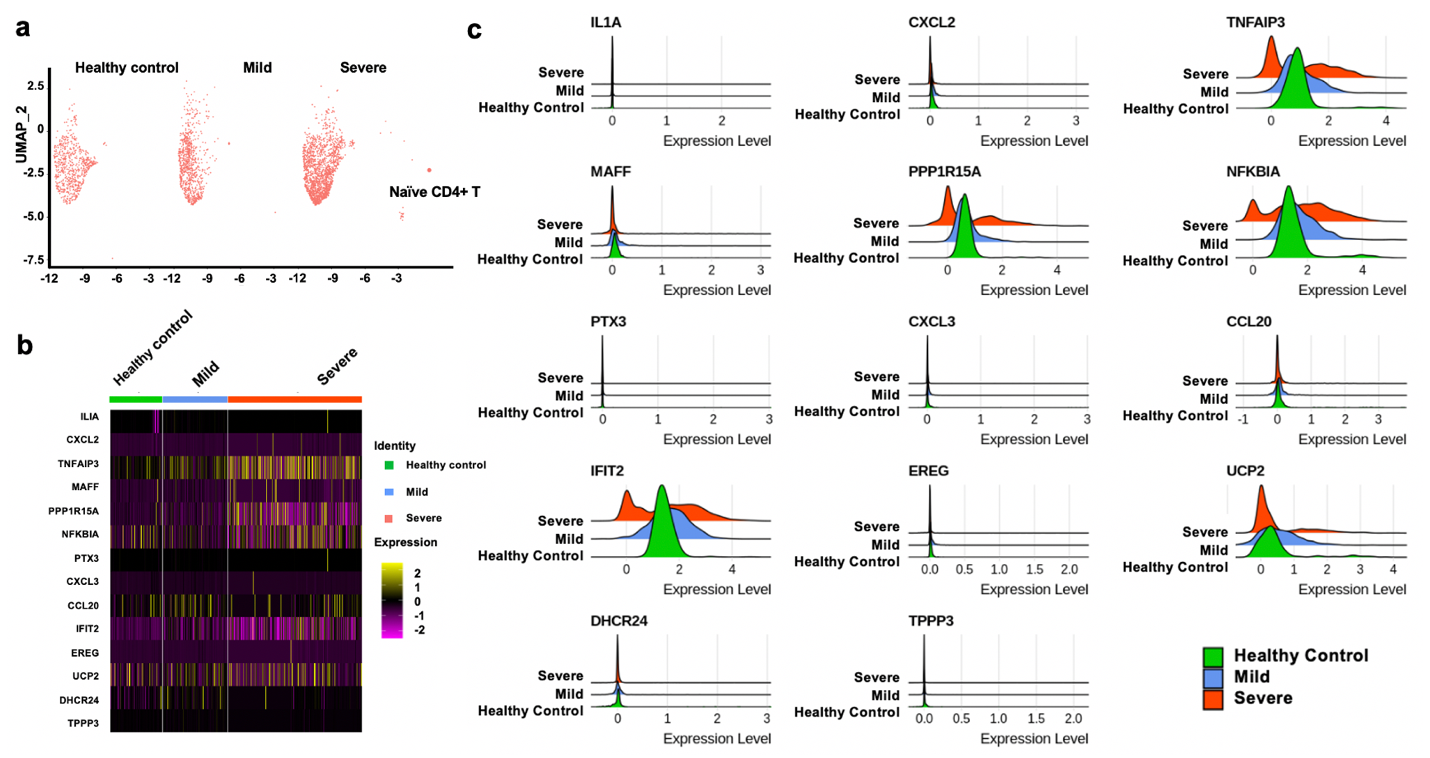


**Fig. S7. Expression of signature genes in naïve CD4+ T cells from patient groups.** **a,** UMAP plots of the naïve CD4+ T cells. Each dot corresponds to one single cell. **b,** Heatmap of fourteen signature genes in three groups. Each vertical bar represents a single cell. Column (cell identity) width is proportional to the number of cells present in that cluster. **c,** Distributions of signature gene expression shown in ridge plots. Red, blue and green colors represent gene expression values in severe, mild patients and healthy controls.


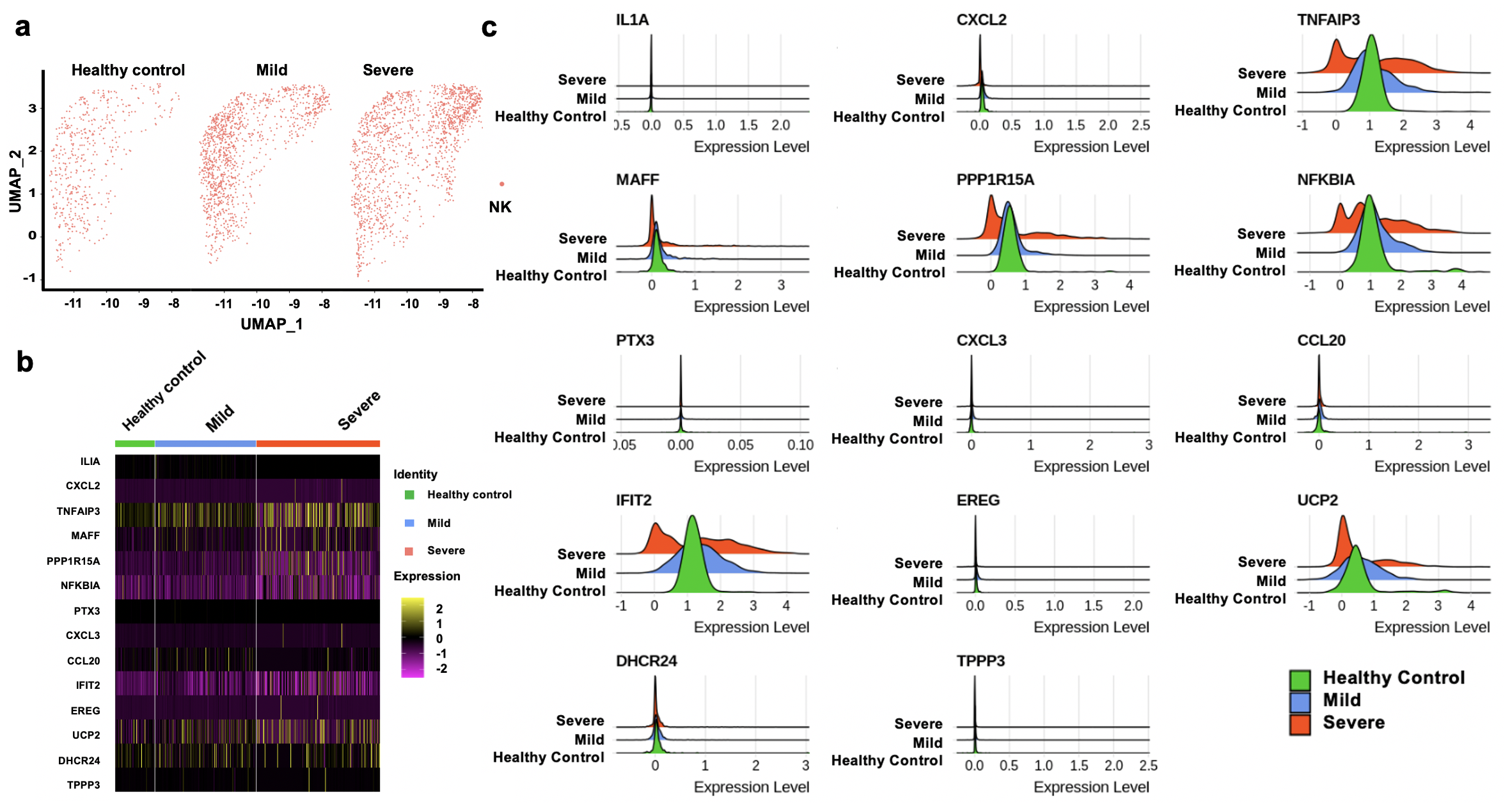


**Fig. S8.** **Expression of signature genes in natural killer (NK) cells from patient groups.** **a,** UMAP plots of the natural killer cells. Each dot corresponds to one single cell. **b,** Heatmap of fourteen signature genes in three groups. Each vertical bar represents a single cell. Column (cell identity) width is proportional to the number of cells present in that cluster. **c,** Distributions of signature gene expression shown in ridge plots. Red, blue and green colors represent gene expression values in severe, mild patients and healthy controls.


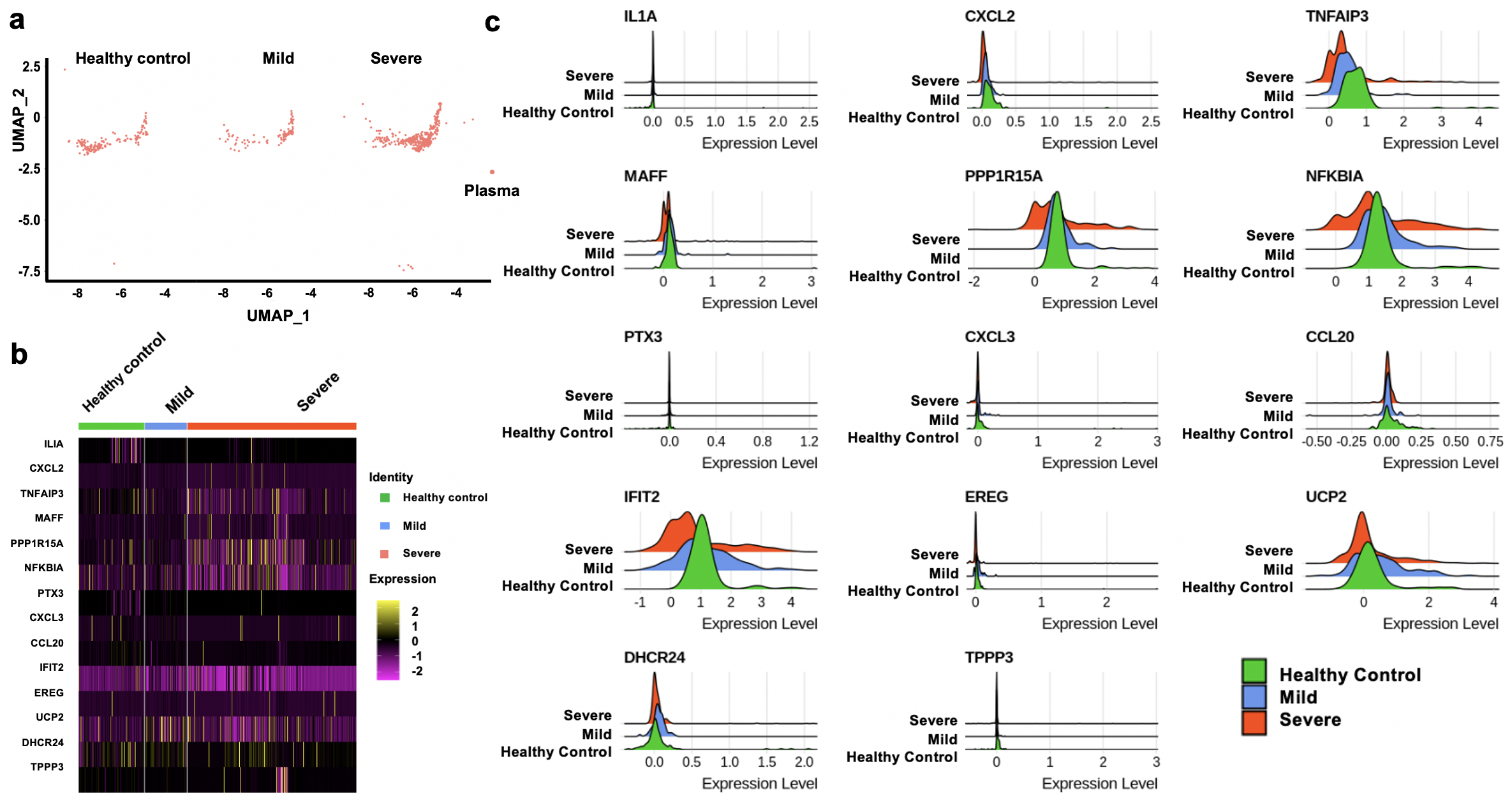


**Figure S9. Expression of signature genes in plasma cells from patient groups.** **a,** UMAP plots of the plasma cells. Each dot corresponds to one single cell. **b,** Heatmap of fourteen signature genes in three groups. Each vertical bar represents a single cell. Column (cell identity) width is proportional to the number of cells present in that cluster. **c,** Distributions of signature gene expression shown in ridge plots. Red, blue and green colors represent gene expression values in severe, mild patients and healthy controls.

**
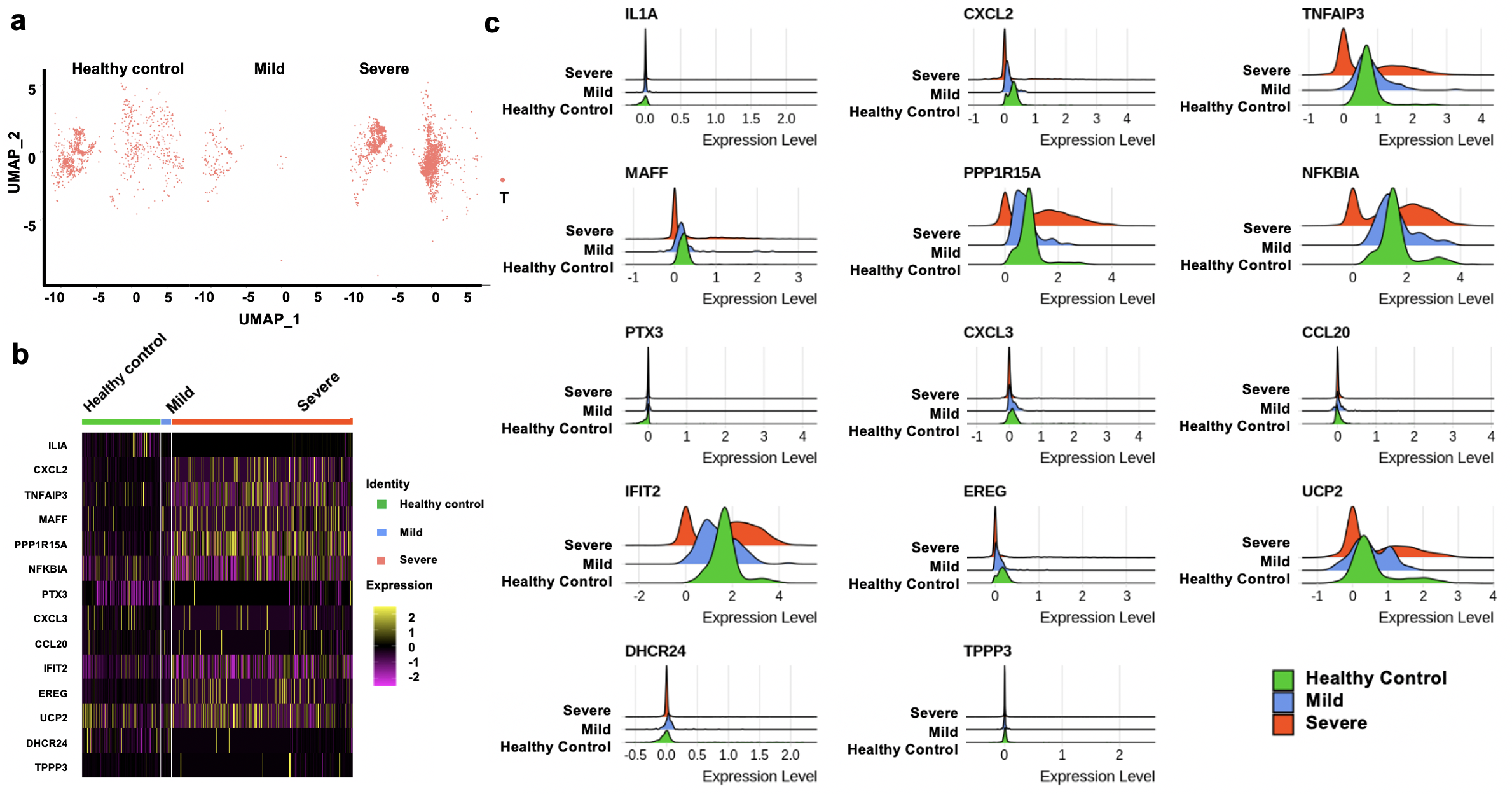
**

**Fig. S10. Expression of signature genes in T cells from patient groups.** **a,** UMAP plots of the T cells. **b,** Heatmap of signature genes in three groups. Each vertical bar represents a single cell. Column (cell identity) width is proportional to the number of cells present in that cluster. **c,** Distributions of gene expression signature were shown in ridge plots. Red, blue and green colors represent gene expression values in severe, mild patients and healthy controls.


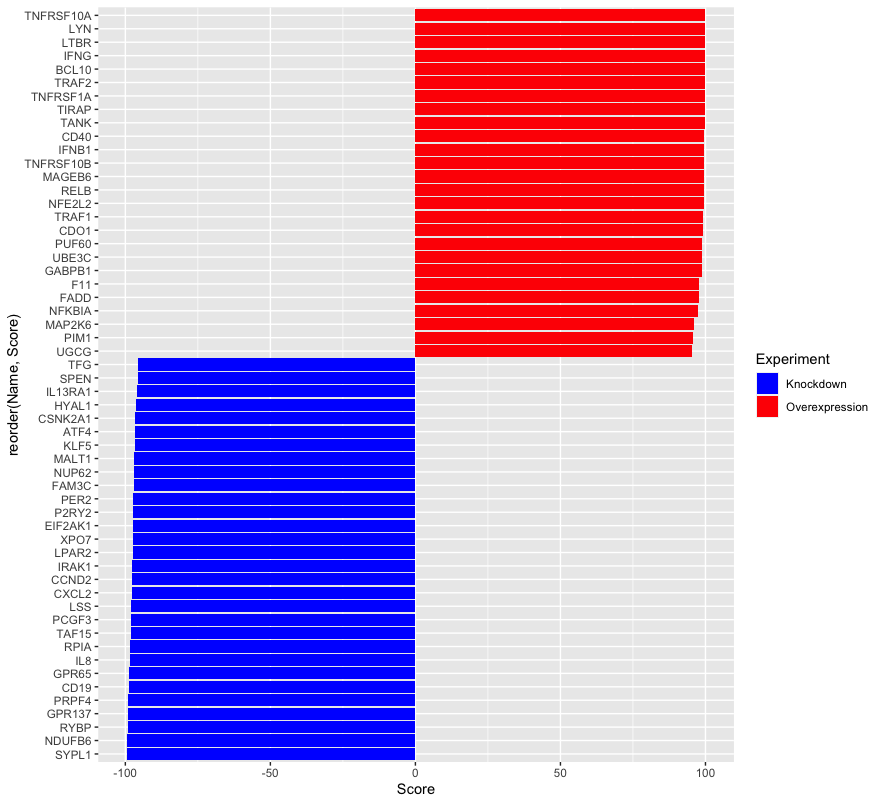


**Fig. S11. Connectivity Scores (CSs) for Genetic perturbations with the 25-gene SARS-CoV-2 Infection Signature.** Red color bars represent CS > 95 and blue color bar represent CS < -95 for gene overexpression and knockdown signatures, respectively.

**
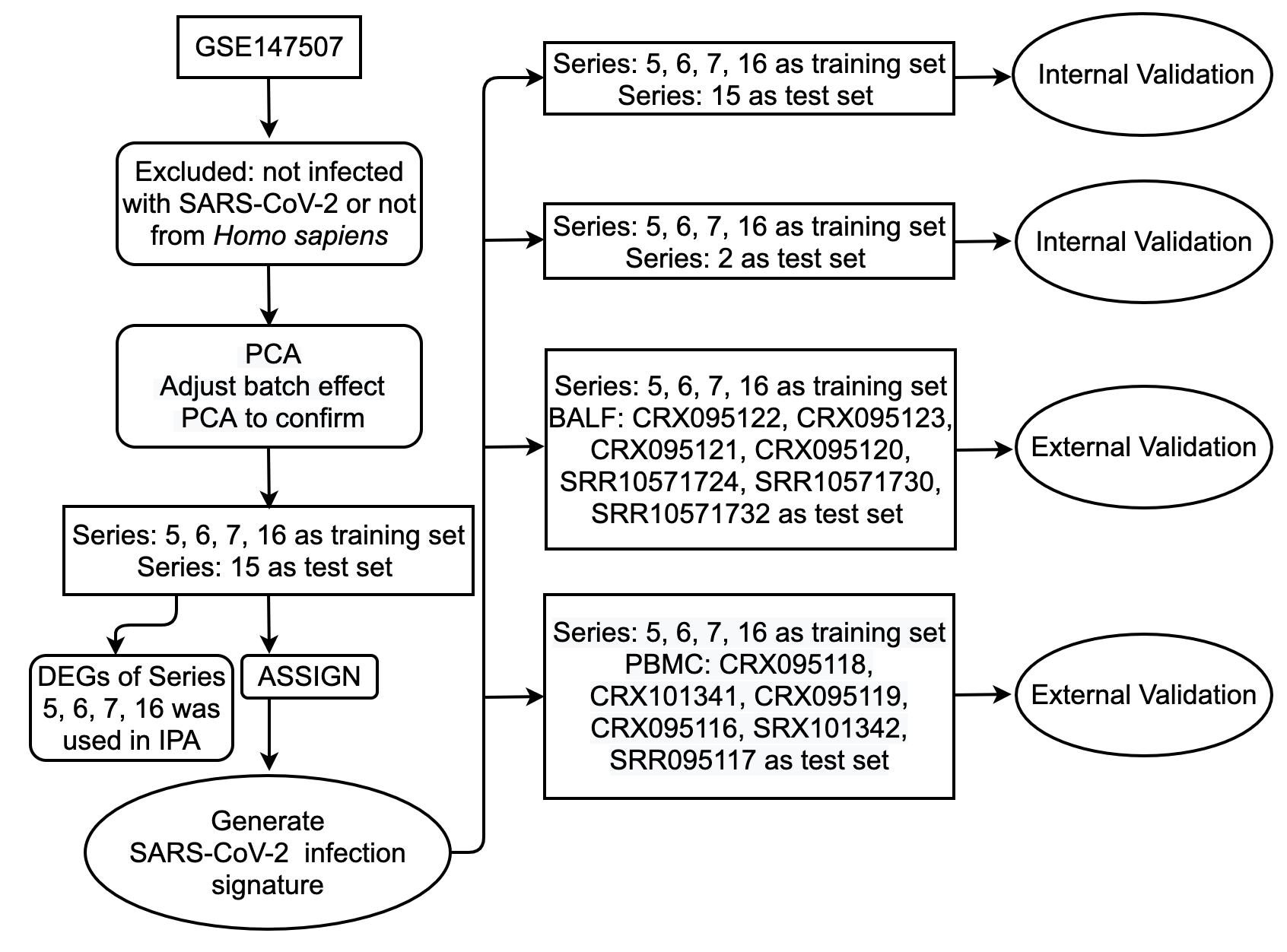
**

**Fig. S12. Data processing steps used in SARS-CoV-2 gene expression signature generation, testing and validation in various datasets.** PCA: principal component analysis, DEGs: differentially expressed genes, IPA: Ingenuity Pathway Analysis, BALF: bronchoalveolar lavage fluid, PBMC: peripheral blood mononuclear cell.

**Supplementary Tables:**

**Table S1**: **Cell markers used to identify cell types in single-cell RNA-Sequencing dataset GSE145926.**

| Cell Type | Cell Marker |
| --- | --- |
| Macrophages  Basal cells | CD68 [1]  S1000A14 [2], KRT6A [3] |
| Dendritic cells  Naive CD4 T cells  Neutrophils  Natural killer cells | IRF7 [4]  IL7R [5]  FCGR3B [6]  CCL5 [7], CD7 [8] |
| Plasma cells | JCHAIN [9] |
| T cells | CD3D [10] |

**Table S2: Selected Connectivity Score (CS) with the 25-gene SARS-CoV-2 infection signature from the ConnectivityMap (CMAP) database.**

|  | CMAP Rank | Score | Type | ID | Name | Description |
| --- | --- | --- | --- | --- | --- | --- |
| 1 | 8559 | -99.37 | kd | CGS001-6856 | SYPL1 | - |
| 2 | 8558 | -99.34 | kd | CGS001-4712 | NDUFB6 | Mitochondrial respiratory chain complex / Complex I |
| 3 | 8557 | -99.12 | cp | BRD-K49865102 | PD-0325901 | MEK inhibitor |
| 4 | 8556 | -99.08 | kd | CGS001-23429 | RYBP | - |
| 5 | 8555 | -99.01 | cp | BRD-K74236984 | UNC-0321 | Histone lysine methyltransferase inhibitor |
| 6 | 8554 | -98.95 | kd | CGS001-56834 | GPR137 | GPCR / Unclassified: 7TM orphan receptors |
| 7 | 8553 | -98.92 | kd | CGS001-9128 | PRPF4 | WD repeat domain containing |
| 8 | 8552 | -98.7 | cp | BRD-K62810658 | PD-98059 | MEK inhibitor |
| 9 | 8550 | -98.69 | cc |  | MEK inhibitor | - |
| 10 | 8551 | -98.69 | kd | CGS001-930 | CD19 | CD molecules |
| 11 | 8549 | -98.66 | kd | CGS001-8477 | GPR65 | GPCR / Class A: Orphans |
| 12 | 8548 | -98.63 | cp | BRD-K12244279 | MEK1-2-inhibitor | MEK inhibitor |
| 13 | 8547 | -98.53 | kd | CGS001-3576 | IL8 | - |
| 14 | 8546 | -98.45 | cp | BRD-K48722833 | iloperidone | Dopamine receptor antagonist |
| 15 | 8545 | -98.37 | kd | CGS001-22934 | RPIA | - |
| 16 | 8544 | -98.31 | cp | BRD-U73238814 | QL-XI-92 | DDR1 inhibitor |
| 17 | 8543 | -98.21 | kd | CGS001-8148 | TAF15 | RNA binding motif (RRM) containing |
| 18 | 8542 | -98.13 | cp | BRD-A11702965 | chromomycin-a3 | DNA binding agent |
| 19 | 8541 | -98 | cp | BRD-U88459701 | atorvastatin | HMGCR inhibitor |
| 20 | 8540 | -97.98 | kd | CGS001-10336 | PCGF3 | Polycomb group ring fingers |
| 21 | 8539 | -97.97 | kd | CGS001-4047 | LSS | Lanosterol biosynthesis pathway |
| 22 | 8538 | -97.87 | kd | CGS001-2920 | CXCL2 | Endogenous ligands |
| 23 | 8537 | -97.63 | kd | CGS001-894 | CCND2 | - |
| 24 | 8536 | -97.59 | cp | BRD-K41895714 | AS-605240 | PI3K inhibitor |
| 25 | 8535 | -97.57 | kd | CGS001-3654 | IRAK1 | Interleukin-1 receptor-associated kinase (IRAK) family |
| 26 | 8534 | -97.53 | kd | CGS001-9170 | LPAR2 | GPCR / Class A: Lysophospholipid receptors: Lysophosphatidic acid |
| 27 | 8533 | -97.37 | kd | CGS001-23039 | XPO7 | Exportins |
| 28 | 8532 | -97.36 | kd | CGS001-27102 | EIF2AK1 | Other PEK family kinases |
| 29 | 8531 | -97.3 | kd | CGS001-5029 | P2RY2 | GPCR / Class A: Purinergic receptors, P2Y |
| 30 | 8530 | -97.29 | kd | CGS001-8864 | PER2 | - |
| 31 | 8529 | -97.18 | kd | CGS001-10447 | FAM3C | - |
| 32 | 8528 | -97.13 | cp | BRD-A13122391 | triptolide | RNA polymerase inhibitor |
| 33 | 8527 | -97.03 | kd | CGS001-23636 | NUP62 | - |
| 34 | 8526 | -96.89 | kd | CGS001-10892 | MALT1 | Immunoglobulin superfamily / Immunoglobulin-like domain containing |
| 35 | 8525 | -96.63 | kd | CGS001-688 | KLF5 | Kruppel-like transcription factors |
| 36 | 8524 | -96.62 | cp | BRD-U51951544 | ZG-10 | JNK inhibitor |
| 37 | 8522 | -96.6 | kd | CGS001-1457 | CSNK2A1 | Casein kinase 2 (CK2) family |
| 38 | 8523 | -96.6 | kd | CGS001-468 | ATF4 | basic leucine zipper proteins |
| 39 | 8521 | -96.48 | cp | BRD-M16762496 | PIK-75 | DNA protein kinase inhibitor |
| 40 | 8520 | -96.43 | cp | BRD-K21680192 | mitoxantrone | Topoisomerase inhibitor |
| 41 | 8519 | -96.36 | kd | CGS001-3373 | HYAL1 | - |
| 42 | 8518 | -96.35 | oe | ccsbBroad304_01061 | NAB2 | - |
| 43 | 8517 | -96.22 | cp | BRD-K53414658 | tivozanib | VEGFR inhibitor |
| 44 | 8516 | -96.02 | kd | CGS001-3597 | IL13RA1 | IL-2 receptor family |
| 45 | 8515 | -95.88 | cp | BRD-K57080016 | selumetinib | MEK inhibitor |
| 46 | 8514 | -95.87 | cp | BRD-K29113274 | ketoconazole | Sterol demethylase inhibitor |
| 47 | 8513 | -95.81 | cp | BRD-K55127134 | fluphenazine | Dopamine receptor antagonist |
| 48 | 8512 | -95.59 | kd | CGS001-23013 | SPEN | RNA binding motif (RRM) containing |
| 49 | 8511 | -95.58 | kd | CGS001-10342 | TFG | - |
| 50 | 8510 | -95.52 | oe | ccsbBroad304_06198 | FCGR2A | CD molecules |
| 51 | 8509 | -95.45 | cp | BRD-K19540840 | saracatinib | SRC inhibitor |
| 52 | 8508 | -95.4 | cp | BRD-K68202742 | trichostatin-a | HDAC inhibitor |
| 53 | 8507 | -95.26 | cp | BRD-K74761218 | WT-171 | HDAC inhibitor |
| 54 | 8506 | -95.17 | cp | BRD-U44618005 | WH-4023 | SRC inhibitor |
| 55 | 8505 | -95.11 | cc |  | SRC inhibitor | - |
| 56 | 8504 | -95.06 | cp | BRD-K14965640 | ibuprofen | Cyclooxygenase inhibitor |
| 57 | 8503 | -94.87 | cp | BRD-K59184148 | SB-216763 | Glycogen synthase kinase inhibitor |
| 58 | 8502 | -94.86 | kd | CGS001-25805 | BAMBI | - |
| 59 | 8501 | -94.8 | kd | CGS001-9124 | PDLIM1 | - |
| 60 | 8500 | -94.72 | kd | CGS001-8835 | SOCS2 | SH2 domain containing |
| 61 | 8499 | -94.44 | kd | CGS001-5526 | PPP2R5B | Serine/threonine phosphatases / Protein phosphatase 2, regulatory subunits |
| 62 | 8498 | -94.27 | kd | CGS001-51135 | IRAK4 | Interleukin-1 receptor-associated kinase (IRAK) family |
| 63 | 8497 | -94.13 | kd | CGS001-5867 | RAB4A | RAB, member RAS oncogene |
| 64 | 8496 | -94.11 | kd | CGS001-9231 | DLG5 | - |
| 65 | 8495 | -93.94 | kd | CGS001-85377 | MICALL1 | - |
| 66 | 8494 | -93.92 | kd | CGS001-22841 | RAB11FIP2 | - |
| 67 | 8493 | -93.72 | kd | CGS001-4352 | MPL | Prolactin receptor family |
| 68 | 8492 | -93.66 | cp | BRD-K15791587 | L-733060 | Tachykinin antagonist |
| 69 | 8491 | -93.59 | kd | CGS001-29775 | CARD10 | - |
| 70 | 8490 | -93.34 | oe | ccsbBroad304_07272 | PPAP2B | Lipid phosphate phosphatases |
| 71 | 8489 | -93.15 | cp | BRD-K81418486 | vorinostat | HDAC inhibitor |
| 72 | 8488 | -93.13 | cp | BRD-K69840642 | ISOX | HDAC inhibitor |
| 73 | 8487 | -93.07 | cp | BRD-A39646320 | HC-toxin | HDAC inhibitor |
| 74 | 8486 | -93.01 | oe | ccsbBroad304_01579 | SOX2 | SRY (sex determining region Y)-boxes |
| 75 | 8485 | -92.96 | cp | BRD-K68756823 | FR-180204 | -666 |
| 76 | 8483 | -92.93 | oe | ccsbBroad304_06847 | RBBP4 | WD repeat domain containing |
| 77 | 8484 | -92.93 | kd | CGS001-80025 | PANK2 | - |
| 78 | 8482 | -92.91 | oe | ccsbBroad304_01540 | SLC2A1 | Solute carriers |
| 79 | 8481 | -92.88 | kd | CGS001-639 | PRDM1 | Zinc fingers, C2H2-type |
| 80 | 8480 | -92.67 | kd | CGS001-3459 | IFNGR1 | Interferon receptor family |
| 81 | 8479 | -92.61 | cc |  | NADH ubiquinone oxidoreductase supernumerary subunits LOF | - |
| 82 | 8478 | -92.58 | kd | CGS001-200734 | SPRED2 | - |
| 83 | 8477 | -92.57 | cp | BRD-K94176593 | TWS-119 | Glycogen synthase kinase inhibitor |
| 84 | 8476 | -92.52 | cp | BRD-A81795050 | U-18666A | Oxidosqualene cyclase inhibitor |
| 85 | 8474 | -92.45 | kd | CGS001-55033 | FKBP14 | EF-hand domain containing |
| 86 | 8475 | -92.45 | kd | CGS001-159 | ADSS | - |
| 87 | 8473 | -92.39 | kd | CGS001-51478 | HSD17B7 | Short chain dehydrogenase/reductase superfamily / Classical SDR fold cluster 2 |
| 88 | 8472 | -92.36 | cp | BRD-K23875128 | RHO-kinase-inhibitor-III [rockout] | Rho associated kinase inhibitor |
| 89 | 8471 | -92.35 | cp | BRD-K52522949 | NCH-51 | HDAC inhibitor |
| 90 | 8470 | -92.34 | cp | BRD-K03319035 | maprotiline | Norepinephrine reuptake inhibitor |
| 91 | 8469 | -92.3 | kd | CGS001-10919 | EHMT2 | Histone methyltransferases |
| 92 | 8468 | -92.24 | cp | BRD-K99964838 | bosutinib | ABL inhibitor |
| 93 | 8467 | -92.15 | kd | CGS001-7625 | ZNF74 | Zinc fingers, C2H2-type |
| 94 | 8466 | -92.02 | kd | CGS001-91137 | SLC25A46 | Miscellaneous SLC25 mitochondrial transporters |
| 95 | 8465 | -92.01 | cc |  | Tricyclic antidepressant | - |
| 96 | 8464 | -92 | kd | CGS001-2589 | GALNT1 | Glycosyltransferase family 2 domain containing |
| 97 | 8463 | -91.97 | cp | BRD-K33583600 | isoliquiritigenin | Guanylate cyclase activator |
| 98 | 8462 | -91.95 | cp | BRD-K89732114 | trifluoperazine | Dopamine receptor antagonist |
| 99 | 8461 | -91.87 | kd | CGS001-2130 | EWSR1 | RNA binding motif (RRM) containing |
| 100 | 8460 | -91.81 | cp | BRD-K61314889 | IWR-1-ENDO | PARP inhibitor |
| 101 | 8459 | -91.73 | cp | BRD-K11558771 | droxinostat | HDAC inhibitor |
| 102 | 8458 | -91.69 | cp | BRD-K85503079 | perospirone | Dopamine receptor antagonist |
| 103 | 8457 | -91.67 | kd | CGS001-7433 | VIPR1 | GPCR / Class B: VIP and PACAP (ADCYAP1) receptors |
| 104 | 8456 | -91.56 | kd | CGS001-6453 | ITSN1 | Rho guanine nucleotide exchange factors |
| 105 | 8455 | -91.43 | cp | BRD-K22503835 | scriptaid | HDAC inhibitor |
| 106 | 8454 | -91.42 | cp | BRD-K80527266 | triacsin-c | Adrenergic receptor antagonist |
| 107 | 8453 | -91.41 | kd | CGS001-65057 | ACD | - |
| 108 | 8452 | -91.39 | oe | ccsbBroad304_00529 | F3 | CD molecules |
| 109 | 8451 | -91.22 | kd | CGS001-8859 | STK19 | G11 family |
| 110 | 8450 | -91.21 | cp | BRD-K50720187 | flupirtine | Glutamate receptor antagonist |
| 111 | 8449 | -91.13 | kd | CGS001-1806 | DPYD | - |
| 112 | 8448 | -91.05 | kd | CGS001-6397 | SEC14L1 | - |
| 113 | 8447 | -91 | kd | CGS001-9918 | NCAPD2 | - |
| 114 | 8446 | -90.75 | kd | CGS001-6794 | STK11 | LKB subfamily |
| 115 | 8445 | -90.56 | cp | BRD-K41160163 | fenobam | Glutamate receptor antagonist |
| 116 | 8444 | -90.44 | cp | BRD-A18579359 | wiskostatin | Neural Wiskott-Aldrich syndrome protein inhibitor |
| 117 | 8443 | -90.41 | kd | CGS001-6767 | ST13 | Tetratricopeptide (TTC) repeat domain containing |
| 118 | 8442 | -90.2 | oe | ccsbBroad304_02138 | TRIP10 | - |
| 119 | 8440 | -90.18 | kd | CGS001-2959 | GTF2B | General transcription factors |
| 120 | 8441 | -90.18 | cp | BRD-A16311756 | profenamine | Butyrylcholinesterase inhibitor |
| 121 | 8439 | -90.05 | kd | CGS001-3030 | HADHA | - |
| 122 | 370 | 90.23 | kd | CGS001-38 | ACAT1 | Lanosterol biosynthesis pathway |
| 123 | 369 | 90.25 | kd | CGS001-51375 | SNX7 | Sorting nexins |
| 124 | 368 | 90.29 | kd | CGS001-9019 | MPZL1 | Immunoglobulin superfamily / V-set domain containing |
| 125 | 367 | 90.31 | kd | CGS001-9296 | ATP6V1F | ATPases / V-type |
| 126 | 366 | 90.33 | kd | CGS001-1955 | MEGF9 | - |
| 127 | 364 | 90.47 | kd | CGS001-10014 | HDAC5 | Histone deacetylases |
| 128 | 362 | 90.68 | kd | CGS001-7511 | XPNPEP1 | Methionyl aminopeptidase |
| 129 | 360 | 90.79 | oe | ccsbBroad304_06681 | P2RY2 | GPCR / Class A: Purinergic receptors, P2Y |
| 130 | 359 | 90.86 | kd | CGS001-5373 | PMM2 | - |
| 131 | 358 | 90.92 | kd | CGS001-29911 | HOOK2 | - |
| 132 | 357 | 90.96 | kd | CGS001-6723 | SRM | - |
| 133 | 356 | 90.97 | oe | ccsbBroad304_07079 | TSPAN4 | Tetraspanins |
| 134 | 353 | 91.13 | oe | ccsbBroad304_06539 | LSS | Lanosterol biosynthesis pathway |
| 135 | 351 | 91.19 | kd | CGS001-6203 | RPS9 | S ribosomal proteins |
| 136 | 350 | 91.21 | kd | CGS001-1019 | CDK4 | Cyclin-dependent kinases, CDK4 subfamily |
| 137 | 348 | 91.23 | kd | CGS001-10482 | NXF1 | - |
| 138 | 349 | 91.23 | kd | CGS001-2936 | GSR | Oxidoreductases |
| 139 | 347 | 91.24 | oe | ccsbBroad304_06648 | CNOT3 | - |
| 140 | 344 | 91.29 | kd | CGS001-51586 | MED15 | - |
| 141 | 342 | 91.34 | kd | CGS001-8894 | EIF2S2 | Serine/threonine phosphatases / Protein phosphatase 1, regulatory subunits |
| 142 | 343 | 91.34 | kd | CGS001-100 | ADA | Adenosine turnover |
| 143 | 340 | 91.37 | kd | CGS001-10681 | GNB5 | WD repeat domain containing |
| 144 | 338 | 91.47 | oe | ccsbBroad304_06753 | POU5F1 | Homeoboxes / POU class |
| 145 | 337 | 91.49 | kd | CGS001-440387 | CTRB2 | - |
| 146 | 335 | 91.53 | oe | ccsbBroad304_04616 | ORMDL3 | - |
| 147 | 336 | 91.53 | kd | CGS001-6584 | SLC22A5 | Organic zwitterions/cation transporters (OCTN) |
| 148 | 333 | 91.69 | kd | CGS001-83743 | GRWD1 | WD repeat domain containing |
| 149 | 332 | 91.75 | oe | ccsbBroad304_01150 | PCCB | Carboxylases |
| 150 | 328 | 91.84 | kd | CGS001-6632 | SNRPD1 | - |
| 151 | 329 | 91.84 | kd | CGS001-4245 | MGAT1 | - |
| 152 | 327 | 91.86 | oe | ccsbBroad304_00272 | CDC25A | Protein tyrosine phosphatases / Class III Cys-based PTPs |
| 153 | 326 | 91.97 | kd | CGS001-2063 | NR2F6 | COUP-TF-like receptors |
| 154 | 325 | 91.98 | kd | CGS001-80351 | TNKS2 | Ankyrin repeat domain containing |
| 155 | 319 | 92.23 | kd | CGS001-5019 | OXCT1 | - |
| 156 | 318 | 92.27 | kd | CGS001-6657 | SOX2 | SRY (sex determining region Y)-boxes |
| 157 | 317 | 92.33 | kd | CGS001-23658 | LSM5 | - |
| 158 | 313 | 92.46 | kd | CGS001-10112 | KIF20A | Kinesins |
| 159 | 312 | 92.49 | kd | CGS001-51727 | CMPK1 | - |
| 160 | 309 | 92.64 | kd | CGS001-6646 | SOAT1 | - |
| 161 | 306 | 92.69 | oe | ccsbBroad304_00477 | LPAR1 | GPCR / Class A: Lysophospholipid receptors: Lysophosphatidic acid |
| 162 | 302 | 92.76 | kd | CGS001-9275 | BCL7B | - |
| 163 | 301 | 92.85 | kd | CGS001-7157 | TP53 | - |
| 164 | 300 | 92.86 | kd | CGS001-10868 | USP20 | Ubiquitin-specific peptidases |
| 165 | 295 | 93.09 | kd | CGS001-7267 | TTC3 | RING-type (C3HC4) zinc fingers |
| 166 | 293 | 93.1 | kd | CGS001-10285 | SMNDC1 | Tudor domain containing |
| 167 | 294 | 93.1 | oe | ccsbBroad304_00602 | SLC37A4 | SLC37 family of phosphosugar/phosphate exchangers |
| 168 | 290 | 93.27 | kd | CGS001-8986 | RPS6KA4 | MSK subfamily |
| 169 | 289 | 93.28 | kd | CGS001-513 | ATP5D | ATPases / F-type |
| 170 | 287 | 93.34 | oe | ccsbBroad304_00088 | FASLG | Tumour necrosis factor (TNF) receptor family |
| 171 | 286 | 93.38 | kd | CGS001-308 | ANXA5 | Annexins |
| 172 | 283 | 93.49 | kd | CGS001-8649 | LAMTOR3 | - |
| 173 | 281 | 93.51 | oe | ccsbBroad304_01186 | PHB | - |
| 174 | 276 | 93.73 | kd | CGS001-8349 | HIST2H2BE | Histones / Replication-dependent |
| 175 | 272 | 93.89 | kd | CGS001-5257 | PHKB | - |
| 176 | 271 | 93.94 | kd | CGS001-8607 | RUVBL1 | ATPases / AAA-type |
| 177 | 270 | 93.96 | kd | CGS001-50814 | NSDHL | Short chain dehydrogenase/reductase superfamily / Extended SDR fold |
| 178 | 269 | 93.98 | kd | CGS001-9308 | CD83 | CD molecules |
| 179 | 267 | 94.03 | kd | CGS001-4191 | MDH2 | - |
| 180 | 264 | 94.12 | oe | ccsbBroad304_03232 | VPS28 | - |
| 181 | 263 | 94.13 | kd | CGS001-64746 | ACBD3 | A-kinase anchor proteins |
| 182 | 260 | 94.27 | kd | CGS001-7272 | TTK | TTK family |
| 183 | 259 | 94.45 | kd | CGS001-8878 | SQSTM1 | - |
| 184 | 258 | 94.46 | oe | ccsbBroad304_00771 | HOXA5 | Homeoboxes / ANTP class: HOXL subclass |
| 185 | 253 | 94.61 | kd | CGS001-2067 | ERCC1 | - |
| 186 | 254 | 94.61 | oe | ccsbBroad304_05390 | DUSP28 | Protein tyrosine phosphatases / Class I Cys-based PTPs: Atypical dual specificity phosphatases |
| 187 | 249 | 94.7 | kd | CGS001-5910 | RAP1GDS1 | Armadillo repeat containing |
| 188 | 246 | 94.82 | kd | CGS001-6772 | STAT1 | SH2 domain containing |
| 189 | 247 | 94.82 | kd | CGS001-9031 | BAZ1B | Zinc fingers, PHD-type |
| 190 | 244 | 94.91 | oe | ccsbBroad304_00879 | IRAK2 | Interleukin-1 receptor-associated kinase (IRAK) family |
| 191 | 243 | 94.93 | kd | CGS001-23161 | SNX13 | Sorting nexins |
| 192 | 242 | 94.99 | kd | CGS001-3326 | HSP90AB1 | Heat shock proteins / HSPC |
| 193 | 241 | 95.02 | kd | CGS001-10298 | PAK4 | PAKB subfamily |
| 194 | 239 | 95.06 | kd | CGS001-501 | ALDH7A1 | Aldehyde dehydrogenases |
| 195 | 238 | 95.08 | kd | CGS001-5981 | RFC1 | ATPases / AAA-type |
| 196 | 235 | 95.18 | kd | CGS001-2853 | GPR31 | GPCR / Class A : Orphans |
| 197 | 233 | 95.2 | kd | CGS001-54472 | TOLLIP | - |
| 198 | 232 | 95.21 | oe | ccsbBroad304_07117 | UGCG | Glycosyltransferase family 2 domain containing |
| 199 | 224 | 95.38 | kd | CGS001-1528 | CYB5A | Cytochrome b genes |
| 200 | 222 | 95.4 | kd | CGS001-2673 | GFPT1 | - |
| 201 | 220 | 95.43 | kd | CGS001-6196 | RPS6KA2 | RSK subfamily |
| 202 | 218 | 95.46 | kd | CGS001-4771 | NF2 | A-kinase anchor proteins |
| 203 | 216 | 95.47 | kd | CGS001-1314 | COPA | Endogenous ligands |
| 204 | 215 | 95.5 | kd | CGS001-283455 | KSR2 | RAF family |
| 205 | 211 | 95.63 | kd | CGS001-11157 | LSM6 | - |
| 206 | 207 | 95.71 | kd | CGS001-29110 | TBK1 | IKK family |
| 207 | 206 | 95.72 | kd | CGS001-79094 | CHAC1 | - |
| 208 | 204 | 95.75 | oe | ccsbBroad304_01203 | PIM1 | PIM family |
| 209 | 200 | 95.82 | kd | CGS001-6938 | TCF12 | Basic helix-loop-helix proteins |
| 210 | 198 | 95.86 | kd | CGS001-2184 | FAH | - |
| 211 | 196 | 95.91 | kd | CGS001-6428 | SRSF3 | RNA binding motif (RRM) containing |
| 212 | 192 | 95.98 | kd | CGS001-10525 | HYOU1 | Heat shock proteins / HSP70 |
| 213 | 191 | 95.99 | kd | CGS001-4726 | NDUFS6 | Mitochondrial respiratory chain complex / Complex I |
| 214 | 187 | 96.11 | kd | CGS001-4323 | MMP14 | Matrix metallopeptidase |
| 215 | 184 | 96.14 | kd | CGS001-1643 | DDB2 | WD repeat domain containing |
| 216 | 183 | 96.16 | oe | ccsbBroad304_01291 | MAP2K6 | MAPKK: STE7 family |
| 217 | 181 | 96.18 | kd | CGS001-9016 | SLC25A14 | Mitochondrial uncoupling proteins |
| 218 | 182 | 96.18 | kd | CGS001-5531 | PPP4C | Serine/threonine phosphatases / Protein phosphatase, catalytic subunits |
| 219 | 179 | 96.24 | kd | CGS001-81550 | TDRD3 | Tudor domain containing |
| 220 | 178 | 96.26 | kd | CGS001-4048 | LTA4H | Hydrolases |
| 221 | 177 | 96.36 | kd | CGS001-204 | AK2 | Adenylate kinases |
| 222 | 173 | 96.42 | kd | CGS001-23200 | ATP11B | Phospholipid-transporting ATPases |
| 223 | 172 | 96.43 | kd | CGS001-4782 | NFIC | - |
| 224 | 169 | 96.49 | kd | CGS001-64170 | CARD9 | - |
| 225 | 164 | 96.6 | kd | CGS001-23012 | STK38L | NDR family |
| 226 | 163 | 96.61 | kd | CGS001-6233 | RPS27A | S ribosomal proteins |
| 227 | 162 | 96.63 | kd | CGS001-79006 | METRN | - |
| 228 | 161 | 96.71 | kd | CGS001-55031 | USP47 | Ubiquitin-specific peptidases |
| 229 | 160 | 96.73 | kd | CGS001-29890 | RBM15B | RNA binding motif (RRM) containing |
| 230 | 157 | 96.83 | kd | CGS001-1871 | E2F3 | - |
| 231 | 156 | 96.89 | kd | CGS001-6259 | RYK | Type XV RTKs: RYK |
| 232 | 154 | 96.93 | kd | CGS001-3125 | HLA-DRB3 | Immunoglobulin superfamily / C1-set domain containing |
| 233 | 150 | 97 | kd | CGS001-8644 | AKR1C3 | Prostaglandin synthases |
| 234 | 146 | 97.08 | kd | CGS001-19 | ABCA1 | ATP binding cassette transporters / subfamily A |
| 235 | 145 | 97.14 | kd | CGS001-9276 | COPB2 | WD repeat domain containing |
| 236 | 139 | 97.31 | oe | ccsbBroad304_01093 | NFKBIA | Ankyrin repeat domain containing |
| 237 | 138 | 97.32 | kd | CGS001-55422 | ZNF331 | Zinc fingers, C2H2-type |
| 238 | 137 | 97.36 | kd | CGS001-3482 | IGF2R | CD molecules |
| 239 | 136 | 97.37 | kd | CGS001-7477 | WNT7B | Endogenous ligands |
| 240 | 133 | 97.51 | kd | CGS001-10381 | TUBB3 | Tubulins |
| 241 | 131 | 97.53 | kd | CGS001-51385 | ZNF589 | Zinc fingers, C2H2-type |
| 242 | 129 | 97.55 | kd | CGS001-57616 | TSHZ3 | Teashirt zinc fingers |
| 243 | 128 | 97.56 | kd | CGS001-5184 | PEPD | Methionyl aminopeptidase |
| 244 | 127 | 97.57 | kd | CGS001-26472 | PPP1R14B | Serine/threonine phosphatases / Protein phosphatase 1, regulatory subunits |
| 245 | 126 | 97.6 | kd | CGS001-7297 | TYK2 | Janus kinase (JakA) family |
| 246 | 124 | 97.7 | oe | ccsbBroad304_02012 | FADD | - |
| 247 | 123 | 97.81 | kd | CGS001-50604 | IL20 | Interleukins and interleukin receptors |
| 248 | 121 | 97.9 | kd | CGS001-25803 | SPDEF | ETS Transcription Factors |
| 249 | 120 | 97.92 | kd | CGS001-1611 | DAP | - |
| 250 | 118 | 97.93 | oe | ccsbBroad304_00532 | F11 | Chymotrypsin |
| 251 | 117 | 98.02 | kd | CGS001-6241 | RRM2 | Ribonucleoside-diphosphate reductases |
| 252 | 114 | 98.08 | kd | CGS001-4267 | CD99 | CD molecules |
| 253 | 111 | 98.11 | kd | CGS001-23224 | SYNE2 | - |
| 254 | 110 | 98.16 | kd | CGS001-50810 | HDGFRP3 | - |
| 255 | 107 | 98.21 | kd | CGS001-1874 | E2F4 | - |
| 256 | 106 | 98.26 | kd | CGS001-5654 | HTRA1 | Serine peptidases / Serine peptidases |
| 257 | 105 | 98.27 | kd | CGS001-10267 | RAMP1 | Receptor (G protein-coupled) activity modifying proteins |
| 258 | 103 | 98.34 | kd | CGS001-22949 | PTGR1 | - |
| 259 | 101 | 98.35 | kd | CGS001-55837 | EAPP | - |
| 260 | 99 | 98.36 | kd | CGS001-55604 | LRRC16A | - |
| 261 | 100 | 98.36 | kd | CGS001-1329 | COX5B | Mitochondrial respiratory chain complex |
| 262 | 372 | 90.05 | cp | BRD-K82823804 | SA-792987 | PKC inhibitor |
| 263 | 371 | 90.21 | cp | BRD-K47150025 | KI-8751 | VEGFR inhibitor |
| 264 | 365 | 90.38 | cp | BRD-K51816706 | oxindole-I | VEGFR inhibitor |
| 265 | 363 | 90.63 | cp | BRD-A38030642 | cyclosporin-a | Calcineurin inhibitor |
| 266 | 361 | 90.68 | cp | BRD-K62353524 | DY-131 | Estrogen receptor agonist |
| 267 | 355 | 91.09 | cp | BRD-K68336408 | tyrphostin-AG-1478 | EGFR inhibitor |
| 268 | 354 | 91.12 | cp | BRD-K78599730 | manumycin-a | Farnesyltransferase inhibitor |
| 269 | 352 | 91.17 | cp | BRD-K16406336 | methylene-blue | Guanylyl cyclase inhibitor |
| 270 | 346 | 91.26 | cp | BRD-K15616905 | CCCP | Mitochondrial oxidative phosphorylation uncoupler |
| 271 | 345 | 91.29 | cp | BRD-K37456065 | VU-0365114-2 | M5 modulator |
| 272 | 341 | 91.36 | cp | BRD-K17075857 | chloroxine | Opioid receptor antagonist |
| 273 | 339 | 91.39 | cp | BRD-A22713669 | BVT-948 | Tyrosine phosphatase inhibitor |
| 274 | 334 | 91.61 | cp | BRD-K60230970 | MG-132 | Proteasome inhibitor |
| 275 | 331 | 91.79 | cp | BRD-K38477985 | malonoben | Protein tyrosine kinase inhibitor |
| 276 | 330 | 91.83 | cp | BRD-K62012036 | acitretin | Retinoid receptor agonist |
| 277 | 324 | 92 | cp | BRD-K66896231 | BRD-K66896231 | Acetylcholinesterase inhibitor |
| 278 | 323 | 92.05 | cp | BRD-K73395020 | SA-1478088 | -666 |
| 279 | 322 | 92.06 | cp | BRD-A11007541 | BCI-hydrochloride | Protein phosphatase inhibitor |
| 280 | 321 | 92.09 | cc |  | PKC inhibitor | - |
| 281 | 320 | 92.14 | cp | BRD-A75517195 | thiazolopyrimidine | CDC inhibitor |
| 282 | 316 | 92.36 | cp | BRD-K28907958 | CD-437 | Retinoid receptor agonist |
| 283 | 315 | 92.37 | cp | BRD-K15600710 | obatoclax | BCL inhibitor |
| 284 | 314 | 92.43 | cp | BRD-K82135108 | elesclomol | Oxidative stress inducer |
| 285 | 311 | 92.53 | cp | BRD-K88677950 | PD-198306 | MAP kinase inhibitor |
| 286 | 310 | 92.55 | cp | BRD-K12184916 | dactolisib | MTOR inhibitor |
| 287 | 308 | 92.64 | cp | BRD-K54233340 | dorsomorphin | AMPK inhibitor |
| 288 | 307 | 92.67 | cp | BRD-K21806131 | tegaserod | Serotonin receptor partial agonist |
| 289 | 304 | 92.71 | cp | BRD-K49810818 | sorafenib | FLT3 inhibitor |
| 290 | 305 | 92.71 | cp | BRD-K77987382 | mebendazole | Tubulin inhibitor |
| 291 | 303 | 92.74 | cp | BRD-A34205397 | suloctidil | Adrenergic receptor antagonist |
| 292 | 299 | 92.87 | cp | BRD-K54095730 | CMPD-1 | p38 MAPK inhibitor |
| 293 | 298 | 92.88 | cc |  | FLT3 inhibitor | - |
| 294 | 297 | 92.92 | cp | BRD-A55484088 | BNTX | Opioid receptor antagonist |
| 295 | 296 | 92.94 | cp | BRD-K39120595 | bithionol | Autotaxin inhibitor |
| 296 | 292 | 93.1 | cp | BRD-A50737080 | CGK-733 | ATR kinase inhibitor |
| 297 | 291 | 93.13 | cp | BRD-A08003242 | rhodomyrtoxin-b | sodium fluorescein uptake inhibitor |
| 298 | 288 | 93.29 | cp | BRD-K03816923 | rottlerin | MAP kinase inhibitor |
| 299 | 285 | 93.48 | cp | BRD-K03109492 | NSC-663284 | CDC inhibitor |
| 300 | 284 | 93.49 | cp | BRD-K35687265 | ON-01910 | PLK inhibitor |
| 301 | 282 | 93.51 | cp | BRD-K26863634 | BIX-01338 | Histone lysine methyltransferase inhibitor |
| 302 | 280 | 93.57 | cp | BRD-K41903098 | diphenoxylate | Opioid receptor agonist |
| 303 | 279 | 93.67 | cp | BRD-K26669427 | WR-216174 | PFMRK inhibitor |
| 304 | 278 | 93.69 | cp | BRD-K13646352 | midostaurin | FLT3 inhibitor |
| 305 | 277 | 93.71 | cp | BRD-K13514097 | everolimus | MTOR inhibitor |
| 306 | 275 | 93.75 | cp | BRD-A80775386 | hyperforin | Cyclooxygenase inhibitor |
| 307 | 273 | 93.87 | cp | BRD-K15409150 | penfluridol | T-type calcium channel blocker |
| 308 | 274 | 93.87 | cp | BRD-K14821540 | FCCP | Mitochondrial oxidative phosphorylation uncoupler |
| 309 | 268 | 94.02 | cp | BRD-K59753975 | vindesine | Tubulin inhibitor |
| 310 | 266 | 94.06 | cp | BRD-K83988098 | alvespimycin | HSP inhibitor |
| 311 | 265 | 94.12 | cp | BRD-K10573841 | tunicamycin | GLCNAC phosphotransferase inhibitor |
| 312 | 262 | 94.2 | cp | BRD-A28105619 | cucurbitacin-i | JAK inhibitor |
| 313 | 261 | 94.22 | cp | BRD-U08759356 | EI-346-erlotinib-analog | EGFR inhibitor |
| 314 | 257 | 94.48 | cp | BRD-K78659596 | MLN-2238 | Proteasome inhibitor |
| 315 | 256 | 94.5 | cp | BRD-K08547377 | irinotecan | Topoisomerase inhibitor |
| 316 | 255 | 94.57 | cp | BRD-K84595254 | strophanthidin | ATPase inhibitor |
| 317 | 252 | 94.67 | cp | BRD-K43405658 | tyrphostin-AG-527 | Protein tyrosine kinase inhibitor |
| 318 | 251 | 94.68 | cp | BRD-A96799240 | 4-hydroxyretinoic-acid | Retinoid receptor binder |
| 319 | 250 | 94.69 | cp | BRD-K15935639 | z-leu3-VS | Proteasome inhibitor |
| 320 | 248 | 94.71 | cp | BRD-K74305673 | IKK-2-inhibitor-V | IKK inhibitor |
| 321 | 245 | 94.89 | cp | BRD-K64517075 | heliomycin | ATP synthase inhibitor |
| 322 | 240 | 95.04 | cp | BRD-K81528515 | nilotinib | ABL inhibitor |
| 323 | 237 | 95.1 | cp | BRD-K26818574 | BIX-01294 | Histone lysine methyltransferase inhibitor |
| 324 | 236 | 95.16 | cp | BRD-K76907295 | VU-0418947-2 | HIF modulator |
| 325 | 234 | 95.18 | cp | BRD-K51318897 | fenbendazole | Tubulin inhibitor |
| 326 | 231 | 95.22 | cp | BRD-A98283014 | calmidazolium | Calcium channel blocker |
| 327 | 230 | 95.24 | cp | BRD-K44432556 | VU-0418946-1 | HIF modulator |
| 328 | 229 | 95.27 | cp | BRD-A82371568 | clofarabine | Ribonucleoside reductase inhibitor |
| 329 | 228 | 95.32 | cc |  | BCL inhibitor | - |
| 330 | 226 | 95.35 | cp | BRD-A45498368 | WYE-125132 | MTOR inhibitor |
| 331 | 227 | 95.35 | cp | BRD-K19295594 | gossypol | BCL inhibitor |
| 332 | 225 | 95.37 | cp | BRD-K89930444 | AG-592 | Tyrosine kinase inhibitor |
| 333 | 223 | 95.38 | cp | BRD-K18518344 | digitoxigenin | ATPase inhibitor |
| 334 | 221 | 95.41 | cp | BRD-K35960502 | niclosamide | DNA replication inhibitor |
| 335 | 219 | 95.44 | cp | BRD-K40255344 | tyrphostin-A9 | Protein tyrosine kinase inhibitor |
| 336 | 217 | 95.46 | cp | BRD-K18619710 | digoxigenin | Steroid |
| 337 | 214 | 95.55 | cp | BRD-K32744045 | disulfiram | Aldehyde dehydrogenase inhibitor |
| 338 | 213 | 95.57 | cp | BRD-A35588707 | teniposide | Topoisomerase inhibitor |
| 339 | 212 | 95.62 | cp | BRD-K95901403 | XL-147 | PI3K inhibitor |
| 340 | 209 | 95.67 | cp | BRD-K17705806 | JTC-801 | Opioid receptor antagonist |
| 341 | 210 | 95.67 | cp | BRD-A55594068 | vinblastine | Microtubule inhibitor |
| 342 | 208 | 95.71 | cp | BRD-K25504083 | cytochalasin-d | Actin polymerization inhibitor |
| 343 | 205 | 95.74 | cp | BRD-K17140735 | SCH-79797 | Proteasome inhibitor |
| 344 | 203 | 95.75 | cp | BRD-K60623809 | SU-11652 | Tyrosine kinase inhibitor |
| 345 | 202 | 95.76 | cp | BRD-K95655893 | MAZ-51 | VEGFR inhibitor |
| 346 | 201 | 95.81 | cp | BRD-K93331255 | lypressin | Vasopressin receptor agonist |
| 347 | 199 | 95.84 | cp | BRD-K78126613 | menadione | Mitochondrial DNA polymerase inhibitor |
| 348 | 197 | 95.88 | cp | BRD-A89434049 | sarmentogenin | ATPase inhibitor |
| 349 | 195 | 95.91 | cp | BRD-A28970875 | puromycin | Protein synthesis inhibitor |
| 350 | 194 | 95.95 | cp | BRD-A02481876 | importazole | Importin-beta transport receptor inhibitor |
| 351 | 193 | 95.96 | cp | BRD-K59469039 | AG-879 | Angiogenesis inhibitor |
| 352 | 190 | 96 | cc |  | Vesicular Transport LOF | - |
| 353 | 189 | 96.04 | cp | BRD-U68942961 | JW-7-24-1 | LCK Inhibitor |
| 354 | 188 | 96.05 | cp | BRD-A34806832 | proscillaridin | ATPase inhibitor |
| 355 | 185 | 96.12 | cp | BRD-A06352508 | SB-218078 | CHK inhibitor |
| 356 | 186 | 96.12 | cp | BRD-A93236127 | digitoxin | ATPase inhibitor |
| 357 | 180 | 96.22 | cp | BRD-K63265447 | docetaxel | Tubulin inhibitor |
| 358 | 175 | 96.37 | cp | BRD-U33728988 | QL-X-138 | MTOR inhibitor |
| 359 | 176 | 96.37 | cp | BRD-A94756469 | digoxin | ATPase inhibitor |
| 360 | 174 | 96.41 | cp | BRD-K21672174 | RO-28-1675 | Glucokinase activator |
| 361 | 171 | 96.44 | cp | BRD-K63606607 | bufalin | ATPase inhibitor |
| 362 | 170 | 96.46 | cp | BRD-K28296557 | AKT-inhibitor-IV | AKT inhibitor |
| 363 | 168 | 96.49 | cp | BRD-A62809825 | thapsigargin | ATPase inhibitor |
| 364 | 167 | 96.55 | cp | BRD-K02526760 | QS-11 | ARFGAP inhibitor |
| 365 | 166 | 96.58 | cp | BRD-A68930007 | ouabain | ATPase inhibitor |
| 366 | 165 | 96.59 | cc |  | IKK inhibitor | - |
| 367 | 158 | 96.82 | cp | BRD-K19894101 | MST-312 | Telomerase inhibitor |
| 368 | 159 | 96.82 | cp | BRD-K04546108 | JAK3-inhibitor-VI | JAK inhibitor |
| 369 | 155 | 96.91 | cc |  | Proteasome inhibitor | - |
| 370 | 152 | 96.93 | cp | BRD-K09638361 | SA-63133 | -666 |
| 371 | 153 | 96.93 | cp | BRD-K65814004 | diphenyleneiodonium | Nitric oxide synthase inhibitor |
| 372 | 151 | 96.98 | cp | BRD-K66792149 | quinoclamine | Algicide |
| 373 | 149 | 97.03 | cp | BRD-A45333398 | periplocymarin | Apoptosis stimulant |
| 374 | 148 | 97.04 | cp | BRD-K26997899 | SA-792574 | Microtubule inhibitor |
| 375 | 147 | 97.07 | cp | BRD-A58564983 | selamectin | Nematocide |
| 376 | 144 | 97.15 | cp | BRD-A80502530 | cinobufagin | ATPase inhibitor |
| 377 | 143 | 97.21 | cp | BRD-M86331534 | pyrvinium-pamoate | AKT inhibitor |
| 378 | 141 | 97.22 | cp | BRD-K12539581 | nocodazole | Tubulin inhibitor |
| 379 | 142 | 97.22 | cp | BRD-K03406345 | azacitidine | DNA methyltransferase inhibitor |
| 380 | 140 | 97.29 | cp | BRD-A47829399 | artesunate | DNA synthesis inhibitor |
| 381 | 135 | 97.39 | cp | BRD-K67506692 | tyrphostin-AG-126 | ERK1 and ERK2 phosphorylation inhibitor |
| 382 | 134 | 97.43 | cp | BRD-K68174511 | torin-2 | MTOR inhibitor |
| 383 | 130 | 97.53 | cp | BRD-K76805682 | SB-415286 | Glycogen synthase kinase inhibitor |
| 384 | 132 | 97.53 | cp | BRD-K47869605 | podophyllotoxin | Microtubule inhibitor |
| 385 | 125 | 97.69 | cc |  | Heat shock 70kDa proteins LOF | - |
| 386 | 122 | 97.88 | cp | BRD-K86003836 | flubendazole | Tubulin inhibitor |
| 387 | 119 | 97.92 | cp | BRD-K24681473 | YM-155 | Survivin inhibitor |
| 388 | 116 | 98.05 | cp | BRD-A63998256 | helveticoside | ATPase inhibitor |
| 389 | 115 | 98.06 | cp | BRD-K91370081 | anisomycin | DNA synthesis inhibitor |
| 390 | 112 | 98.1 | cp | BRD-K99498722 | NPI-2358 | Tubulin inhibitor |
| 391 | 113 | 98.1 | cp | BRD-A54927599 | KF-38789 | P-selectin inhibitor |
| 392 | 109 | 98.17 | cp | BRD-K47983010 | BX-795 | IKK inhibitor |
| 393 | 108 | 98.2 | cp | BRD-A47513740 | calyculin | Protein phosphatase inhibitor |
| 394 | 104 | 98.31 | cp | BRD-A26002865 | verrucarin-a | Protein synthesis inhibitor |
| 395 | 102 | 98.34 | cc |  | HIF activator | - |
| 396 | 98 | 98.41 | cp | BRD-K56334280 | amonafide | Topoisomerase inhibitor |
| 397 | 97 | 98.45 | cp | BRD-K82109576 | vincristine | Tubulin inhibitor |
| 398 | 96 | 98.47 | cp | BRD-K98490050 | amsacrine | Topoisomerase inhibitor |
| 399 | 95 | 98.49 | kd | CGS001-11331 | PHB2 | - |
| 400 | 93 | 98.52 | cc |  | ATPase inhibitor | - |
| 401 | 94 | 98.52 | cp | BRD-A62184259 | cycloheximide | Protein synthesis inhibitor |
| 402 | 92 | 98.53 | kd | CGS001-11057 | ABHD2 | Abhydrolase domain containing |
| 403 | 91 | 98.56 | cp | BRD-K53972329 | ruxolitinib | JAK inhibitor |
| 404 | 90 | 98.61 | kd | CGS001-6461 | SHB | SH2 domain containing |
| 405 | 89 | 98.63 | kd | CGS001-6909 | TBX2 | T-boxes |
| 406 | 88 | 98.65 | kd | CGS001-7311 | UBA52 | L ribosomal proteins |
| 407 | 87 | 98.66 | kd | CGS001-3309 | HSPA5 | Heat shock proteins / HSP70 |
| 408 | 86 | 98.68 | kd | CGS001-4723 | NDUFV1 | Mitochondrial respiratory chain complex / Complex I |
| 409 | 85 | 98.69 | kd | CGS001-332 | BIRC5 | Inhibitors of apoptosis (IAP) protein family |
| 410 | 83 | 98.7 | cp | BRD-U86922168 | QL-XII-47 | BTK inhibitor |
| 411 | 84 | 98.7 | cp | BRD-K06792661 | narciclasine | Coflilin signaling pathway activator |
| 412 | 82 | 98.71 | kd | CGS001-2879 | GPX4 | - |
| 413 | 81 | 98.73 | cp | BRD-K80348542 | cephaeline | Protein synthesis inhibitor |
| 414 | 80 | 98.77 | cp | BRD-K92138166 | mammea-a | another antibiotic |
| 415 | 79 | 98.8 | cp | BRD-K76674262 | homoharringtonine | Protein synthesis inhibitor |
| 416 | 77 | 98.82 | kd | CGS001-6129 | RPL7 | L ribosomal proteins |
| 417 | 78 | 98.82 | kd | CGS001-9020 | MAP3K14 | MAPKKK: STE-unique family |
| 418 | 76 | 98.83 | kd | CGS001-4713 | NDUFB7 | Mitochondrial respiratory chain complex / Complex I |
| 419 | 75 | 98.84 | kd | CGS001-5050 | PAFAH1B3 | - |
| 420 | 74 | 98.87 | cp | BRD-A15010982 | HU-211 | Glutamate receptor antagonist |
| 421 | 72 | 98.89 | oe | ccsbBroad304_00606 | GABPB1 | Ankyrin repeat domain containing |
| 422 | 73 | 98.89 | oe | ccsbBroad304_11405 | UBE3C | - |
| 423 | 68 | 98.91 | cc |  | Tubulin inhibitor | - |
| 424 | 69 | 98.91 | cp | BRD-A36707673 | hydroxycholesterol | LXR agonist |
| 425 | 70 | 98.91 | cp | BRD-K80431395 | triciribine | AKT inhibitor |
| 426 | 71 | 98.91 | cp | BRD-A25687296 | emetine | Protein synthesis inhibitor |
| 427 | 67 | 98.92 | kd | CGS001-64764 | CREB3L2 | basic leucine zipper proteins |
| 428 | 66 | 98.94 | oe | ccsbBroad304_02691 | PUF60 | RNA binding motif (RRM) containing |
| 429 | 65 | 98.95 | kd | CGS001-79080 | CCDC86 | - |
| 430 | 64 | 99.01 | cp | BRD-K28143534 | cyproheptadine | Histamine receptor antagonist |
| 431 | 62 | 99.03 | kd | CGS001-51160 | VPS28 | - |
| 432 | 63 | 99.03 | kd | CGS001-100133941 | CD24 | CD molecules |
| 433 | 61 | 99.05 | oe | ccsbBroad304_05981 | CDO1 | - |
| 434 | 59 | 99.11 | kd | CGS001-79170 | PRR15L | - |
| 435 | 60 | 99.11 | kd | CGS001-51053 | GMNN | - |
| 436 | 57 | 99.13 | kd | CGS001-79724 | ZNF768 | Zinc fingers, C2H2-type |
| 437 | 58 | 99.13 | cp | BRD-K49669041 | BX-912 | Pyruvate dehydrogenase kinase inhibitor |
| 438 | 55 | 99.17 | kd | CGS001-9326 | ZNHIT3 | Zinc fingers, HIT-type |
| 439 | 56 | 99.17 | kd | CGS001-11004 | KIF2C | Kinesins |
| 440 | 54 | 99.18 | kd | CGS001-11065 | UBE2C | Ubiquitin-conjugating enzymes E2 |
| 441 | 53 | 99.19 | cp | BRD-A15079084 | phorbol-12-myristate-13-acetate | PKC activator |
| 442 | 52 | 99.22 | cp | BRD-K94325918 | kinetin-riboside | Apoptosis stimulant |
| 443 | 51 | 99.26 | oe | ccsbBroad304_07097 | TRAF1 | - |
| 444 | 50 | 99.3 | cp | BRD-K04853698 | LDN-193189 | Serine/threonine kinase inhibitor |
| 445 | 49 | 99.32 | kd | CGS001-1212 | CLTB | - |
| 446 | 48 | 99.35 | oe | ccsbBroad304_01090 | NFE2L2 | basic leucine zipper proteins |
| 447 | 47 | 99.39 | kd | CGS001-8767 | RIPK2 | Receptor interacting protein kinase (RIPK) family |
| 448 | 46 | 99.41 | kd | CGS001-11284 | PNKP | - |
| 449 | 45 | 99.44 | oe | ccsbBroad304_01388 | RELB | NFkappaB transcription factor family |
| 450 | 44 | 99.45 | kd | CGS001-221692 | PHACTR1 | Phosphatase and actin regulators |
| 451 | 43 | 99.46 | kd | CGS001-2021 | ENDOG | - |
| 452 | 39 | 99.47 | kd | CGS001-291 | SLC25A4 | Mitochondrial nucleotide transporter subfamily |
| 453 | 40 | 99.47 | kd | CGS001-51116 | MRPS2 | Mitochondrial ribosomal proteins / small subunits |
| 454 | 41 | 99.47 | kd | CGS001-1182 | CLCN3 | Ion channels / Chloride channels : Voltage-sensitive |
| 455 | 42 | 99.47 | kd | CGS001-978 | CDA | - |
| 456 | 37 | 99.49 | oe | ccsbBroad304_07304 | TNFRSF10B | Tumour necrosis factor (TNF) receptor family |
| 457 | 38 | 99.49 | oe | ccsbBroad304_05098 | MAGEB6 | - |
| 458 | 36 | 99.5 | kd | CGS001-7480 | WNT10B | Wingless-type MMTV integration sites |
| 459 | 35 | 99.51 | kd | CGS001-7187 | TRAF3 | - |
| 460 | 33 | 99.53 | kd | CGS001-4795 | NFKBIL1 | - |
| 461 | 34 | 99.53 | kd | CGS001-54566 | EPB41L4B | - |
| 462 | 31 | 99.54 | cp | BRD-K91145395 | prostratin | PKC activator |
| 463 | 32 | 99.54 | cp | BRD-A52650764 | ingenol | PKC activator |
| 464 | 30 | 99.55 | cc |  | Protein synthesis inhibitor | - |
| 465 | 28 | 99.58 | oe | ccsbBroad304_00832 | IFNB1 | Interferons |
| 466 | 29 | 99.58 | kd | CGS001-1677 | DFFB | - |
| 467 | 26 | 99.61 | kd | CGS001-58477 | SRPRB | - |
| 468 | 27 | 99.61 | kd | CGS001-51071 | DERA | - |
| 469 | 23 | 99.63 | oe | ccsbBroad304_00259 | CD40 | Tumour necrosis factor (TNF) receptor family |
| 470 | 24 | 99.63 | kd | CGS001-7494 | XBP1 | basic leucine zipper proteins |
| 471 | 25 | 99.63 | kd | CGS001-8738 | CRADD | - |
| 472 | 22 | 99.68 | oe | ccsbBroad304_07531 | TANK | - |
| 473 | 21 | 99.71 | kd | CGS001-59286 | UBL5 | - |
| 474 | 17 | 99.72 | cc |  | PKC activator | - |
| 475 | 18 | 99.72 | oe | ccsbBroad304_01710 | TRAF2 | RING-type (C3HC4) zinc fingers |
| 476 | 19 | 99.72 | oe | ccsbBroad304_01688 | TNFRSF1A | Tumour necrosis factor (TNF) receptor family |
| 477 | 20 | 99.72 | oe | ccsbBroad304_09396 | TIRAP | - |
| 478 | 16 | 99.74 | kd | CGS001-7184 | HSP90B1 | Heat shock proteins / HSPC |
| 479 | 13 | 99.77 | oe | ccsbBroad304_00833 | IFNG | Interferons |
| 480 | 14 | 99.77 | oe | ccsbBroad304_02048 | BCL10 | - |
| 481 | 15 | 99.77 | oe | ccsbBroad304_06542 | LTBR | Tumor necrosis factor receptor superfamily |
| 482 | 11 | 99.79 | kd | CGS001-4907 | NT5E | Adenosine turnover |
| 483 | 12 | 99.79 | kd | CGS001-1054 | CEBPG | basic leucine zipper proteins |
| 484 | 10 | 99.81 | oe | ccsbBroad304_00954 | LYN | Src family |
| 485 | 9 | 99.82 | kd | CGS001-3516 | RBPJ | - |
| 486 | 7 | 99.84 | kd | CGS001-4151 | MB | - |
| 487 | 8 | 99.84 | kd | CGS001-383 | ARG1 | Arginase |
| 488 | 6 | 99.86 | oe | ccsbBroad304_07306 | TNFRSF10A | Tumour necrosis factor (TNF) receptor family |
| 489 | 4 | 99.89 | kd | CGS001-22937 | SCAP | WD repeat domain containing |
| 490 | 5 | 99.89 | kd | CGS001-83694 | RPS6KL1 | RSKL family |
| 491 | 3 | 99.91 | cc |  | NFKB Activation GOF | - |
| 492 | 2 | 99.92 | kd | CGS001-694 | BTG1 | - |
| 493 | 1 | 99.95 | kd | CGS001-10113 | PREB | WD repeat domain containing |
| 494 | 8373 | -82.94 | cp | BRD-K51485625 | ritonavir | HIV protease inhibitor |
| 495 | 8264 | -72.2 | cp | BRD-K04210847 | tamoxifen | Estrogen receptor antagonist |
| 496 | 7248 | -13.59 | cp | BRD-A96255180 | ribavirin | Antiviral |
| 497 | 7209 | -12.76 | cp | BRD-K92731339 | perindopril | ACE inhibitor |
| 498 | 6910 | -7.41 | cp | BRD-K60038276 | irbesartan | Angiotensin receptor antagonist |
| 499 | 6829 | -6.34 | cp | BRD-K14791739 | fluticasone | Glucocorticoid receptor agonist |
| 500 | 6621 | -3.99 | cp | BRD-K99451608 | lopinavir | HIV protease inhibitor |
| 501 | 6436 | -2.71 | cp | BRD-K78485176 | olmesartan | Angiotensin receptor antagonist |
| 502 | 6362 | -2.36 | cp | BRD-K76205745 | losartan | Angiotensin receptor antagonist |
| 503 | 6135 | -1.34 | cp | BRD-A91699651 | chloroquine | Antimalarial |
| 504 | 5870 | -0.19 | cp | BRD-K89348303 | ramipril | ACE inhibitor |
| 505 | 4633 | 0.42 | cp | BRD-K62310379 | fluticasone | Glucocorticoid receptor agonist |
| 506 | 4515 | 0.88 | cp | BRD-K83144676 | olmesartan | Angiotensin antagonist |
| 507 | 3914 | 2.88 | cp | BRD-A93424738 | dexamethasone | Glucocorticoid receptor agonist |
| 508 | 2889 | 10.36 | cp | BRD-A35108200 | dexamethasone | Glucocorticoid receptor agonist |
| 509 | 2761 | 12.12 | cp | BRD-A17655518 | ibuprofen | Cyclooxygenase inhibitor |
| 510 | 565 | 81.09 | cp | BRD-A48570745 | ivermectin | GABA receptor agonist |
| List of all strong connectivity scores (CS) from CMAP with SARS-CoV-2 signature. CS <= -90 or CS >= 90 were considered strong connections. All strong connections and many proposed small molecules for COVID-19 treatment are listed. kd, cp, cc, and oe indicate gene knockdown, CMAP class, CMAP compound and gene overexpression, respectively. | | | | | | |

**Table S3: Description of the samples from GSE147507 used for SARS-CoV-2 signature generation and internal validation [11].**

| Cell Type  (number of samples) | Cell Line | Series | Not infected with SARS-CoV-2 | SARS-CoV-2 Infection  Multiplicity of infection (MOI)  Time point | Training/Testing set |
| --- | --- | --- | --- | --- | --- |
| Lung Adenocarcinoma (6) | A549 | 5 | A549 Mock | A549 SARS-CoV-2 (MOI 2)  24 hours post-treatment | Training |
| Lung Adenocarcinoma (6) | A549 overexpressing human *ACE2* | 6 | A549-*ACE2* Mock | A549-ACE2 SARS-CoV-2 (MOI 0.2)  24 hours post-treatment | Training |
| Lung Adenocarcinoma (6) | Calu-3 | 7 | Calu-3 Mock | Calu-3 SARS-CoV-2 (MOI 2)  24 hours post-treatment | Training |
| Lung Adenocarcinoma (6) | A549 overexpressing human *ACE2* | 16 | A549-*ACE2* Mock | A549-ACE2 SARS-CoV-2 (MOI 2)  24shours post-treatment | Training |
| Lung adenocarcinoma (6) | A549 | 2 | A549 Mock | A549 SARS-CoV-2 (MOI 0.2)  24 hours post-treatment | Testing |
| COVID19 Lung Patient Sample (4) | Lung Biopsy | 15 | Healthy Lung Biopsy | Postmortem COVID-19 patients | Testing |

**Table S4: Description of the external validation human datasets used for SARS-CoV-2 signature [11].**

| Datasets | Cell Type (Number of Samples) | Accession Number | |
| --- | --- | --- | --- |
|  |  | Healthy Control | COVID-19 Patients |
| CRA002390, SRR10571724, SRR10571730, SRR10571732 | Bronchoalveolar lavage fluid cells (7) | SRR10571724, SRR10571730, SRR10571732 | CRX095120, CRX095121, CRX095122, CRX095123 |
|  |  |  |  |
| CRA002390 | Peripheral blood mononuclear cells (6) | CRX101341, CRX101342, CRX095116 | CRX095117, CRX095118, CRX095119 |
|  |  |  |  |

**References**

1. Liao, M. *et al.* Single-cell landscape of bronchoalveolar immune cells in patients with COVID-19. *Nat. Med.* **26**, 842–844 (2020).

2. He, H. *et al.* 12-O-tetradecanoylphorbol-13-acetate promotes breast cancer cell motility by increasing S100A14 Level in a Kruppel-like transcription factor 4 (KLF4)-dependent manner. *J. Biol. Chem.* **289**, 9089–9099 (2014).

3. Biton, A. *et al.* Independent Component Analysis Uncovers the Landscape of the Bladder Tumor Transcriptome and Reveals Insights into Luminal and Basal Subtypes. *Cell Rep.* **9**, 1235–1245 (2014).

4. Jang, J. S. *et al.* Rsad2 is necessary for mouse dendritic cell maturation via the IRF7-mediated signaling pathway. *Cell Death Dis.* **9**, (2018).

5. Heron, M. *et al.* Variation in IL7R predisposes to sarcoid inflammation. *Genes Immun.* **10**, 647–653 (2009).

6. Thomas, H. B., Moots, R. J., Edwards, S. W. & Wright, H. L. Whose gene is it anyway? the effect of preparation purity on neutrophil transcriptome studies. *PLoS One* **10**, 1–15 (2015).

7. Ottaviani, C. *et al.* CD56brightCD16- NK cells accumulate in psoriatic skin in response to CXCL10 and CCL5 and exacerbate skin inflammation. *Eur. J. Immunol.* **36**, 118–128 (2006).

8. Cupedo, T. *et al.* Human fetal lymphoid tissue-inducer cells are interleukin 17-producing precursors to RORC+ CD127+ natural killer-like cells. *Nat. Immunol.* **10**, 66–74 (2009).

9. Harper, S. J. *et al.* Expression of J chain mRNA in duodenal IgA plasma cells in IgA nephropathy. *Kidney Int.* **45**, 836–844 (1994).

10. Noutsias, M. *et al.* Expression of functional T-cell markers and T-cell receptor Vbeta repertoire in endomyocardial biopsies from patients presenting with acute myocarditis and dilated cardiomyopathy. *Eur. J. Heart Fail.* **13**, 611–618 (2011).

11. Cavalli, E. *et al.* Transcriptomic analysis of COVID‑19 lungs and bronchoalveolar lavage fluid samples reveals predominant B cell activation responses to infection. *Int. J. Mol. Med.* 1266–1273 (2020) doi:10.3892/ijmm.2020.4702.
